# Artificial van der Waals hybrid synapse and its application to acoustic pattern recognition

**DOI:** 10.1038/s41467-020-17849-3

**Published:** 2020-08-07

**Authors:** Seunghwan Seo, Beom-Seok Kang, Je-Jun Lee, Hyo-Jun Ryu, Sungjun Kim, Hyeongjun Kim, Seyong Oh, Jaewoo Shim, Keun Heo, Saeroonter Oh, Jin-Hong Park

**Affiliations:** 1 Department of Electrical and Computer Engineering, Sungkyunkwan University, Suwon, 16419 Korea; 2 Semiconductor R&D Center, Samsung Electronics Co. Ltd, Hwasung, 18448 Korea; 3 Foundry Division, Samsung Electronics Co. Ltd., Youngin, 17113 Korea; 4 Department of Mechanical Engineering, Massachusetts Institute of Technology (MIT), Cambridge, MA 02139 USA; 5 Division of Electrical Engineering, Hanyang University, Ansan, 15588 Korea; 6 Sungkyunkwan Advanced Institute of Nanotechnology (SAINT), Sungkyunkwan University, Suwon, 16417 Korea

**Keywords:** Electronic devices, Electronic devices

## Abstract

Brain-inspired parallel computing, which is typically performed using a hardware neural-network platform consisting of numerous artificial synapses, is a promising technology for effectively handling large amounts of informational data. However, the reported nonlinear and asymmetric conductance-update characteristics of artificial synapses prevent a hardware neural-network from delivering the same high-level training and inference accuracies as those delivered by a software neural-network. Here, we developed an artificial van-der-Waals hybrid synapse that features linear and symmetric conductance-update characteristics. Tungsten diselenide and molybdenum disulfide channels were used selectively to potentiate and depress conductance. Subsequently, via training and inference simulation, we demonstrated the feasibility of our hybrid synapse toward a hardware neural-network and also delivered high recognition rates that were comparable to those delivered using a software neural-network. This simulation involving the use of acoustic patterns was performed with a neural network that was theoretically formed with the characteristics of the hybrid synapses.

## Abstract (web summary)

Designing high-performance and energy efficient neural network hardware remains a challenge. Here, the authors develop a van der Waals hybrid synaptic device that features linear and symmetric conductance-update characteristics and demonstrate the feasibility for hardware neural network performing acoustic pattern recognition.

## Introduction

It is predicted that a large amount of unstructured data in the upcoming Big Data era will not be processed efficiently via conventional serial computing technology based on the von Neumann architecture^1^. Thus, a brain-inspired parallel computing technology suitable for dealing with such unstructured data was recently proposed^2,3^. Brain-inspired computing is generally performed using a hardware neural-network (HW-NN) platform consisting of numerous artificial synapses^4,5^. Therefore, considerable effort has been directed toward the implementation of artificial synapses mimicking the behavior of biological synapses, such as short-term plasticity and long-term plasticity^6,7^. Synaptic devices based on various operating mechanisms and materials have been reported, including resistive random-access memory (RRAM), phase-change memory (PCM), field-effect transistors (FETs) with ferroelectric or charge-trapping layer, electrochemical memory, optoelectronic memory^8–20^. However, it has not been demonstrated that an HW-NN composed of such synaptic devices can perform training and inference tasks with the same level of accuracy as a software-based neural-network (SW-NN). This is because such devices do not sufficiently satisfy the synaptic characteristics, such as the cycle-to-cycle variation (CCV), device-to-device variation (DDV), retention time, endurance, number of conductance states, dynamic range, and linear/symmetrical conductance change^20–23^. In particular, the linearity and symmetricity of the conductance change are known to significantly affect the inference accuracy after the training process of HW-NNs^24,25^.

J. Woo et al. and S. Park et al. reported HfO_x_ RRAM and AlO_x_/TiN PCMO-based synaptic devices, respectively, where the nonlinear and asymmetrical conductance change resulted in a low inference accuracy of <40% for the Modified National Institute of Standards and Technology (MNIST) dataset^26,27^. For the HfO_x_ RRAM, an AlO_x_ barrier layer was introduced, consequently improving its conductance change more linearly, but this device inherently presented low dynamic range and high CCV^18,26^. For the hafnium-zirconium oxide FeFET-type synapse reported by M. Jerry et al., a highly symmetric conductance change was achieved, leading to a high inference accuracy of 90% for the MNIST dataset^28^. However, in the FeFET synapse, non-identical spikes were applied for controlling the conductance state. This is because most of the reported synaptic devices operate on the basis of a physical mechanism that cannot change the conductance linearly with respect to the applied voltage. Meanwhile, E. J. Fuller et al. reported an ionic floating gate (IFG)-based synaptic device featuring a very linear conductance change that was achieved by a gradual composition modulation in the IFG^29^. Recently, various studies to approach the training/inference accuracy of an SW-NN have been attempted by designing synaptic unit cells with highly linear and symmetric conductance change characteristic^24,30,31^. S. Kim et al. reported a very linear conductance change in their synaptic unit cell consisting of three transistors and one capacitor^30^. Although the excellent linearity allowed an accurate training process, the volatility of the cell made inference based on the trained information difficult. S. Ambrogio et al. and X. Sun et al. applied nonvolatile memory elements, such as PCM and ferroelectric capacitors, to their synapse cells, yielding high training and inference accuracies simultaneously^24,31^. However, such synapse cells require highly complex peripheral circuits for operation, as well as a large number of devices. Therefore, additional studies on artificial synapses should be performed to achieve desirable synaptic characteristics required for high-performance HW-NNs.

Herein, we report an artificial vdW-hybrid synaptic device that features linear and symmetric conductance change characteristics. The excellent conductance controllability is accomplished by using tungsten diselenide (WSe_2_) and molybdenum disulfide (MoS_2_) hybrid channels, which are specialized for linear conductance potentiation and depression, respectively. We also discuss the CCV & DDV, relative standard deviation (RSD), endurance, symmetricity, and dynamic range for the long-term potentiation (LTP)/long-term depression (LTD) characteristic curves with respect to the conditions of weight control spikes. In particular, our synaptic device is investigated and compared with other devices reported heretofore, in terms of various synaptic characteristics mentioned above, weight updating energy, and active area (see Supplementary Table 1). Finally, we demonstrate the feasibility of the vdW-hybrid synaptic device for an HW-NN and present high recognition rates close to those for an SW-NN via training and inference simulation, where both our designed acoustic patterns and existing MNIST digit patterns are used.

## Results

### Artificial van der Waals hybrid synapse

**Fig. 1:**
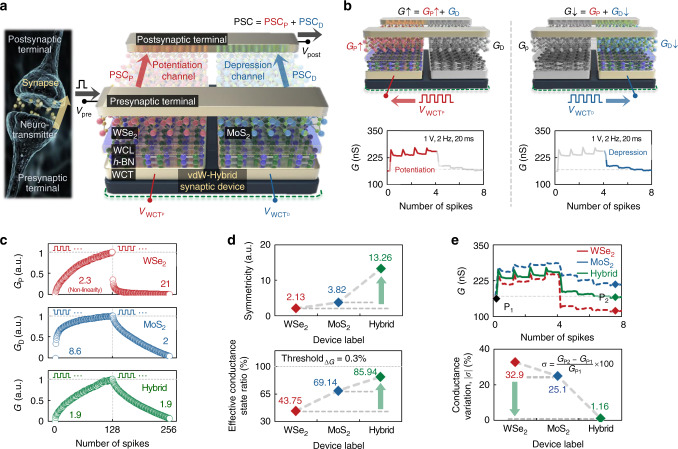
vdW-hybrid synaptic device with excellent controllability of the conductance. **a** Functional and structural comparison of the biological synapse with the vdW-hybrid synaptic device. **b** Demonstration of potentiation and depression operations with four spikes. **c** Long-term potentiation (LTP) and depression (LTD) curves including extracted nonlinearity values for the control devices (WSe_2_ and MoS_2_ synaptic devices) and the vdW-hybrid synaptic device, where 128 excitatory and inhibitory spikes were applied consecutively to the WCTs. **d** Symmetricity and effective conductance-state ratio (threshold_Δ*G*_ = 0.3%) extracted from the LTP/LTD curves. **e** Conductance responses when eight spikes were applied in a row (four excitatory and four inhibitory) to the WCTs of the three types of devices, and extracted conductance variations |σ|.

Biological synapses are known to transmit spike signals from the presynaptic terminal to the postsynaptic terminal using neurotransmitters and to adjust their synaptic weights on the basis of the timing of the spike signals^32^. In this study, as shown in Fig. 1a, we implemented an vdW-hybrid synaptic device that successfully mimics the operation of biological synapses and presented excellent synaptic characteristics. This vdW-hybrid synaptic device features two signal paths for potentiation and depression operations, which are formed on WSe_2_ (for hole transport)/hexagonal-boron nitride (*h*-BN) and MoS_2_ (for electron transport)/*h*-BN heterostructures, respectively. Here, such vdW heterostructures are free from concerns about lattice mismatching owing to the dangling-bond-free surface nature of the vdW materials^33–35^, thereby allowing the formation of interfacial defect-free floating gate structure^36,37^ or the modulation of the number of interfacial traps/dipoles for achieving the synaptic functionalities^12,18,38^. The potentiation and depression channels are tied by two electrodes, which are defined as the presynaptic and postsynaptic terminals, and the two channels have an individual gate electrode functioning as a weight control terminal (WCT). Additional information regarding the vdW-hybrid synaptic device, such as an optical microscopy image, thickness profiles of vdW materials confirmed via atomic force microscopy (AFM), and the Raman spectra of each vdW material are provided in Supplementary Fig. 1. When a voltage spike (*V*_pre_) is applied to the presynaptic terminal, a postsynaptic current (PSC) appears at the postsynaptic terminal, which is the sum of the PSCs of the potentiation (PSC_P_) and depression (PSC_D_) channels (PSC = PSC_P_ + PSC_D_). This indicates that the conductance of the vdW-hybrid synaptic device (*G*) is identical to the sum of the conductance values of the potentiation (*G*_P_) and depression (*G*_D_) channels (*G* = *G*_P_ + *G*_D_). Therefore, as shown in Fig. 1b, the synaptic conductance of this device can be potentiated (*G*↑ = *G*_P_↑ + *G*_D_) or depressed (*G*↓ = *G*_P_ + *G*_D_↓) by applying only a positive voltage spike (+*V*_WCT_) to the WCT. The conductance of the WSe_2_ and MoS_2_ channels is modulated on the basis of the phenomenon that electrons are only trapped in the weight control layer (WCL) formed on *h*-BN under the positive voltage spike condition. This differs from the conventional transistor-type synapse, where both trapping and detrapping of electrons are used for potentiation and depression, which allows highly symmetric operation of the synapse^12,39^. For accurate operation of the vdW-hybrid synaptic device, two different polarity FET devices with a similar current level are needed to be integrated. When voltage spikes with an amplitude of 1 V, a duration of 20 ms, and a frequency of 2 Hz were applied to the WCT for the potentiation channel four times consecutively, the conductance of the vdW-hybrid synaptic device increased in steps from 159 to 257 nS (potentiation operation). When the same voltage spikes were applied to the WCT for the depression channel, the conductance decreased in steps to 163 nS, which was similar to the initial conductance value (depression operation).

Following the demonstration of the selective controllability of the conductance in the vdW-hybrid synaptic device, we compared its synaptic characteristics with those of a control synaptic device with a WSe_2_ or MoS_2_ channel. As shown in Fig. 1c, we confirmed the LTP/LTD characteristics in each synaptic device, where 128 excitatory and inhibitory voltage spikes were applied in a row to the WCTs of the devices. For the WSe_2_ synaptic device, the conductance increased linearly and decreased nonlinearly (red curve). The conductance change of the MoS_2_ synaptic device (blue curve) exhibited the opposite behavior. According to these results, the channels composed of WSe_2_ and MoS_2_ were specialized for potentiation and depression, respectively. Therefore, in the vdW-hybrid synaptic device that selectively exploits the specialized channels for the LTP/LTD, the conductance states are uniformly distributed (green curve). The measurement setup for the synaptic devices is described in detail in Supplementary Fig. 2. To evaluate the LTP/LTD characteristics quantitatively, we extracted the nonlinearity from the characteristic curves (β_P_ from the LTP curve and β_D_ from the LTD curve). The nonlinearity values (β_P_/β_D_) of the WSe_2_ and MoS_2_ synaptic devices were 2.3/21 and 8.6/2, respectively. For the vdW-hybrid synaptic device, the nonlinearity values were 1.9/1.9. The dynamic ranges of the WSe_2_, MoS_2_, and vdW-hybrid synaptic devices, which were defined as the difference between *G*_max_ and *G*_min_ (*G*_max_ – *G*_min_), were 237, 210, and 191 nS, respectively. These nonlinearity values were analyzed and compared with the values of previously reported artificial synapses, as shown in Supplementary Fig. 3. We then calculated the symmetricity indicating the degree of symmetry between the LTP and LTD characteristic curves in Fig. 1d (top). Details regarding the calculation are provided in Supplementary Fig. 4. The symmetricity values of the WSe_2_ and MoS_2_ synaptic devices were approximately 2.13 and 3.82, respectively, and the symmetricity was improved to 13.26 for the vdW-hybrid channel. Here, there are flake-to-flake variations in the vdW channels in terms of defect density and doping concentration, which consequently affect the synaptic characteristics including the linearity and symmetricity of the LTP and LTD curves. Detailed analysis regarding with this issue is provided in Supplementary Fig. 5. In addition, the nonlinearity and symmetricity values were investigated in multiple samples to confirm the DDV in the nonlinearity and symmetricity, as shown in Supplementary Fig. 6. Furthermore, we determined and compared the effective conductance-state ratios, as shown in Fig. 1d (bottom), because an insufficient conductance change (Δ*G*) in the LTP and LTD curves has no effect on the recognition rate in the HW-NN. The effective conductance-state ratio was defined as the ratio of the number of conductance states in which Δ*G* exceeded a certain percentage of *G*_max_/*G*_min_ (threshold_Δ*G*_) to the total number of conductance states. When threshold_Δ*G*_ was set as 0.3%, the WSe_2_ and MoS_2_ synaptic devices exhibited effective conductance-state ratios of 43.75% and 69.14%, respectively, and the vdW-hybrid synaptic device exhibited a relatively high ratio of 85.94%. As shown in Fig. 1e, we applied eight spikes in a row for potentiation and depression (four excitatory spikes and four inhibitory spikes) to the WCTs of the three devices. Consequently, the conductance of the WSe_2_ and MoS_2_ devices decreased (conductance variation σ = –32.9%) and increased (σ = +25.1%), respectively, compared with the initial values. For the hybrid synaptic device, a conductance similar to the initial value was observed under the same spike conditions (σ = +1.16%). Here, the conductance variation σ was calculated using the equation shown in Fig. 1e (bottom). Additional results measured under different combinations of eight spikes are provided in Supplementary Fig. 7, (e.g., two excitatory spikes, two inhibitory spikes, two excitatory spikes, and two inhibitory spikes).

### Analysis of vdW-hybrid synaptic device

**Fig. 2:**
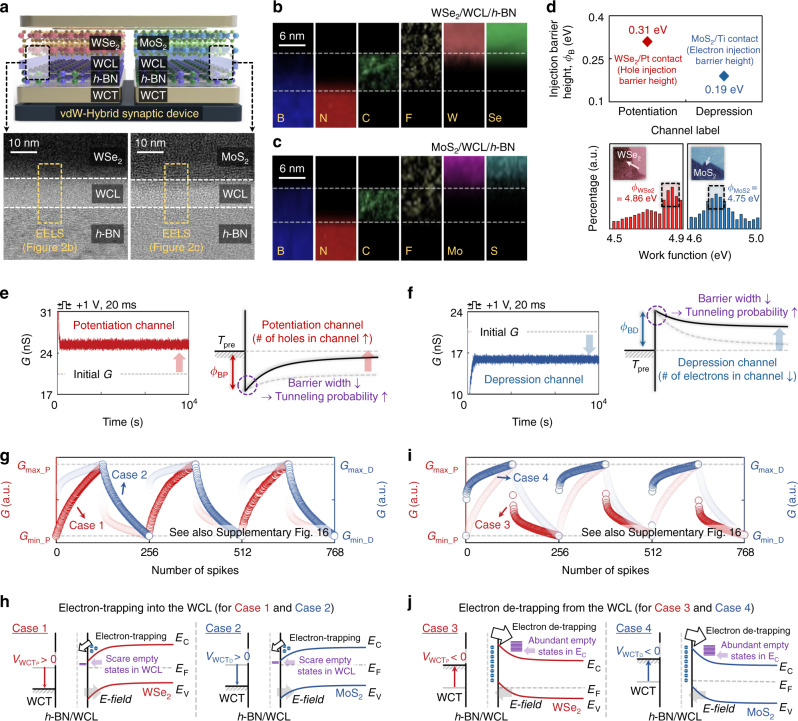
Study on the weight control mechanism of the vdW-hybrid synaptic device. **a** X-TEM images of the WSe_2_/WCL/*h*-BN (potentiation channel) and MoS_2_/WCL/*h*-BN (depression channel) structures. **b**, **c** EELS mapping images obtained on the cross-sectional regions. **d** Extracted injection barrier height and work function for the potentiation and depression channels. **e**, **f** Excitatory (**e**) and inhibitory (**f**) PSC in the potentiation and depression channels. Illustration of energy band diagrams when an excitatory spike was applied to the WCTs for the potentiation and depression channels. **g** LTP curves through the WSe_2_ channel (Case 1) and LTD curves through the MoS_2_ channel (Case 2), where 128 excitatory spikes were applied in a row to the WCTs. **h** Illustration of energy band diagrams describing phenomena of electron trapping into the WCL. **i** LTP curves through MoS_2_ channel (Case 3) and LTD curves through WSe_2_ channel (Case 4), where 128 inhibitory spikes were applied in a row to the WCTs. **j** Illustration of energy band diagrams describing phenomena of electron detrapping from the WCL.

The excellent conductance controllability of the vdW-hybrid synaptic device was due to the electron-trapping phenomenon in the WCL, which was formed by exposing CF_4_ plasma on top of the *h*-BN (details are presented in the METHODS Section). As shown in Fig. 2a, we examined the WCL via cross-sectional transmission electron microscopy (X-TEM). By performing CF_4_ plasma treatment with a reactive ion etcher power of 10 W and a process time of 10 s, the WCL was created at depths of 11.1 and 10.5 nm from the surface underneath the potentiation and depression channels, respectively. The regions inside the yellow dotted line in Fig. 2a were analyzed via electron energy-loss spectroscopy (EELS) mapping, yielding atomic-composition information for each region. As shown in Fig. 2b and c, the WCL regions mainly exhibited signals corresponding to C (green), F (yellow), and B (weak signal, blue). As expected, signals corresponding to W (bright red), Se (bright green), Mo (purple), and S (cyan) were obtained in the regions of WSe_2_ and MoS_2_. The B (blue) and N (red) signals also appeared in the region of *h*-BN. As shown in Supplementary Figs. 8, 9, and 10, we analyzed the energy distribution of the WCL/*h*-BN via micro photoluminescence and micro X-ray photoelectron spectroscopy measurements^40–42^. Additionally, we estimated the trap density in the WCL region containing C and F atoms, obtaining density values of 5.2 × 10^17^ and 5.8 × 10^17^ cm^–3^ for the potentiation and depression channels, respectively. These trap-density values were on the same order as the number of electrons stored in the floating gate of current flash memory cell, as discussed in Supplementary Fig. 11 (http://www.itrs2.net/). As depicted in Fig. 2d, we investigated carrier injection barrier heights and work functions for the WSe_2_ (potentiation) and MoS_2_ (depression) channels via the modified Richardson plotting method, based on a thermionic emission-diffusion model and Kelvin probe force microscopy (KPFM) analysis, respectively (see also Supplementary Fig. 12 and Note 8)^43,44^. A hole barrier height of 0.31 eV and work function of 4.86 were estimated for the WSe_2_ channel, where a platinum contact is formed between the pre/postsynaptic terminals and channel. Meanwhile, an electron barrier height of 0.19 eV and work function of 4.75 eV were obtained for the MoS_2_ channel, where a titanium contact is formed between the pre/postsynaptic terminals and channel. Therefore, the WSe_2_ (potentiation) and MoS_2_ (depression) channels were confirmed as p- and n-type channels, respectively. We then investigated the spike responses for the current flow through the potentiation and depression channels in detail. When an excitatory voltage spike was applied to the WCTs for the WSe_2_ and MoS_2_ channels, the PSCs flowing through the potentiation and depression channels increased from 20.2 to 25.6 nA and decreased from 21.1 to 16.1 nA for 10^4^ seconds, denoting that the trap states of WCL have high confinement energy (see Fig. 2d). This is because the electrons trapped in the WCL increased and decreased the number of holes and electrons in the WSe_2_ and MoS_2_ channels, respectively, decreasing and increasing the width of the tunneling barrier (*W*_TN_) from the presynaptic terminal (*T*_pre_) metal to the vdW channels, as shown in Fig. 2d (bottom). When an inhibitory spike was applied, as shown in Supplementary Fig. 13, the PSC decreased and increased in the potentiation and depression channels, respectively. The operating energy for reading and updating a weight were approximately from 0.12 to 0.71 nJ (for reading energy) and 0.79 (for updating energy in the potentiation channel)/0.93 (for updating energy in the depression channel) pJ, respectively, where a spike with 1 V of amplitude and 10 ms of duration was applied^45^. Such dissipated energy per event were determined by *P=I×V×t*_duration_^12,16,18^, and relevant details are provided in Supplementary Fig. 14. The PSC responses with respect to the amplitude and duration of the spike and with respect to the conditions of CF_4_ plasma treatment were also investigated, as shown in Supplementary Fig. 15. As shown in Fig. 2e, when excitatory spikes with a 1-V amplitude, 20-ms duration, and 2-Hz frequency were applied consecutively to the WCTs underneath the WSe_2_ and MoS_2_ channels, the conductance linearly increased (Case 1) and decreased (Case 2), respectively. Under the excitatory-spike condition, the energy band of the semiconductor near the WCL was instantly bent downward, generating an electric field (*E*) that attracted electrons toward the WCL (see Fig. 2f). Simultaneously, the probability of trap states being filled increased because the Fermi level of the semiconductor was close to its conduction band edge. Therefore, the trap sites at the WCL were partially filled with electrons during each excitatory spike, which gradually changed the conductance of the potentiation and depression channels. In contrast, as shown in Fig. 2g, when the same inhibitory voltage spikes were applied in a row, the conductance nonlinearly decreased and increased at the WSe_2_ and MoS_2_ channels, respectively. The energy band of the semiconductor near the WCL was instantly bent upward when the inhibitory spike was applied, generating an electric field for electron detrapping (see Fig. 2h). Additionally, because there were many empty states within the conduction band of the semiconductor, which were well aligned with the filled trap sites in the energy level, most of the trapped electrons were released from the WCL during the initial few inhibitory spikes. Consequently, when the inhibitory spikes were applied in a row after the excitatory spikes, the conductance rapidly changed at the beginning stages and then gradually became saturated. In the proposed hybrid synapse device, the linearity of PSC was determined by (i) the PSC updating origin (electron trapping into the WCL or detrapping from the WCL), and (ii) the polarity of the channel (p- or n-type channel) (see Supplementary Fig. 16). We further investigated the spike response for the synaptic device using an MoSe_2_ that normally functions as an n-type channel and subsequently confirmed the similar LTP/LTD characteristics of the MoSe_2_ and MoS_2_ synaptic devices (see Supplementary Fig. 17). Meanwhile, the nonlinearity for the potentiation and depression channels was investigated with respect to (i) the amplitudes and durations of the spikes, and (ii) the conditions of CF_4_ plasma treatment, as shown in Supplementary Fig. 18. The information on the spike responses for the potentiation and depression channels without WCL is provided in Supplementary Fig. 19.

### Synaptic characteristics of the vdW-hybrid synaptic device with respect to various spike conditions

**Fig. 3:**
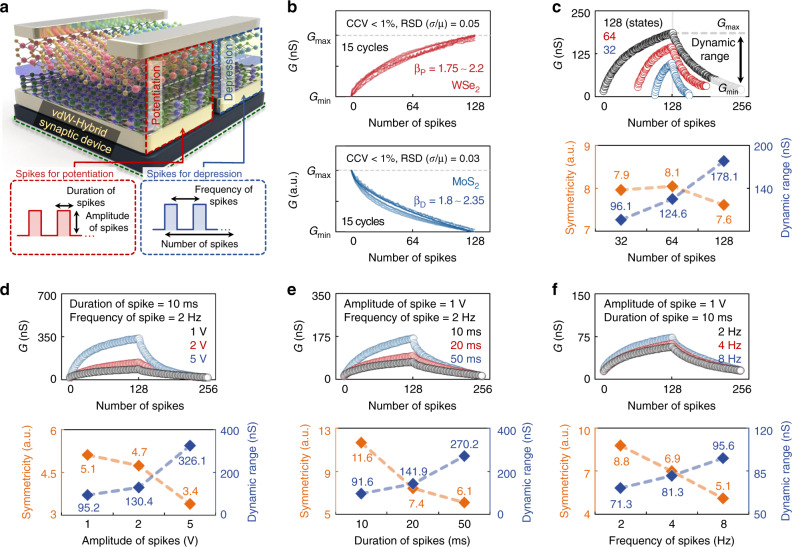
Characteristics of the vdW-hybrid synaptic device with respect to various spike conditions. **a** Synaptic device schematics including explanation on spike condition. **b** LTP and LTD curves for 15 cycles and evaluated CCV and RSD (σ/μ) values for the potentiation and depression channels. **c**–**f** Extracted symmetricity and dynamic range from the LTP/LTD curves measured under various spike conditions, such as the number of spikes (32–128) (**c**), the amplitude of spikes (1–5 V) (**d**), the duration of spikes (10–50 ms) (**e**), and the frequency of spikes (2–8 Hz) (**f**).

Next, we examined the CCV and RSD for the LTP/LTD curves and the symmetricity and dynamic range of the vdW-hybrid device under various voltage spike conditions. These indices significantly affect the performance of an HW-NN composed of artificial synapses^20,23,46^. Figure 3a explains the two voltage spike conditions—one for potentiation and the other for depression—where the number, amplitude, duration, and frequency of the pulses were varied. As shown in Fig. 3b, we measured the LTP/LTD characteristics 15 times and then evaluated the CCV and RSD^21,46^. The CCV was estimated as <1% in the LTP/LTD curves, as shown in Supplementary Fig. 20. Here, the nonlinearity ranging from 1.75 to 2.2 for the potentiation channel and from 1.8 to 2.35 for the depression channel was confirmed. For the RSD, which represents the ratio of the standard deviation (σ) to the mean (μ), values of 0.05 and 0.03 were obtained in the LTP and LTD curves, respectively. Also, as shown in Supplementary Fig. 21, we investigated the endurance (>10^5^ weight updating, 500 cycles of LTP/LTD) of the vdW-hybrid device. We then extracted the symmetricity and dynamic range from the LTP/LTD curves obtained when 32, 64, and 128 voltage spikes were applied, as shown in Fig. 3c. While the symmetricity was not significantly affected by the number of spikes, the dynamic range increased rapidly as the number of spikes increased (symmetricity/dynamic range: 7.95/96.12 nS for 32 states, 8.05/124.6 nS for 64 states, and 7.61/178 nS for 128 states). In addition to the effects of the number of spikes, we investigated the symmetricity and the dynamic range under different spike voltages. As the amplitude of the spikes increased from 1 to 5 V, the symmetricity decreased from 5.11 to 3.39, and the dynamic range increased from 95 to 326 nS (see Fig. 3d). As the duration of the spikes increased from 10 to 50 ms, the symmetricity decreased from 11.65 to 6.11, and the dynamic range increased from 91 to 270 nS (see Fig. 3e). As the frequency of the spikes increased from 2 to 8 Hz, the symmetricity decreased from 8.79 to 5.11, and the dynamic range increased from 71 to 95 nS (see Fig. 3f). As the amplitude, duration, and frequency of the spikes increased, the symmetricity was degraded and the dynamic range value increased, indicating that these performance indices had a tradeoff relationship with each other. A detailed analysis of the results for the symmetricity and the dynamic range is provided in Supplementary Fig. 22.

### Acoustic pattern recognition task

**Fig. 4:**
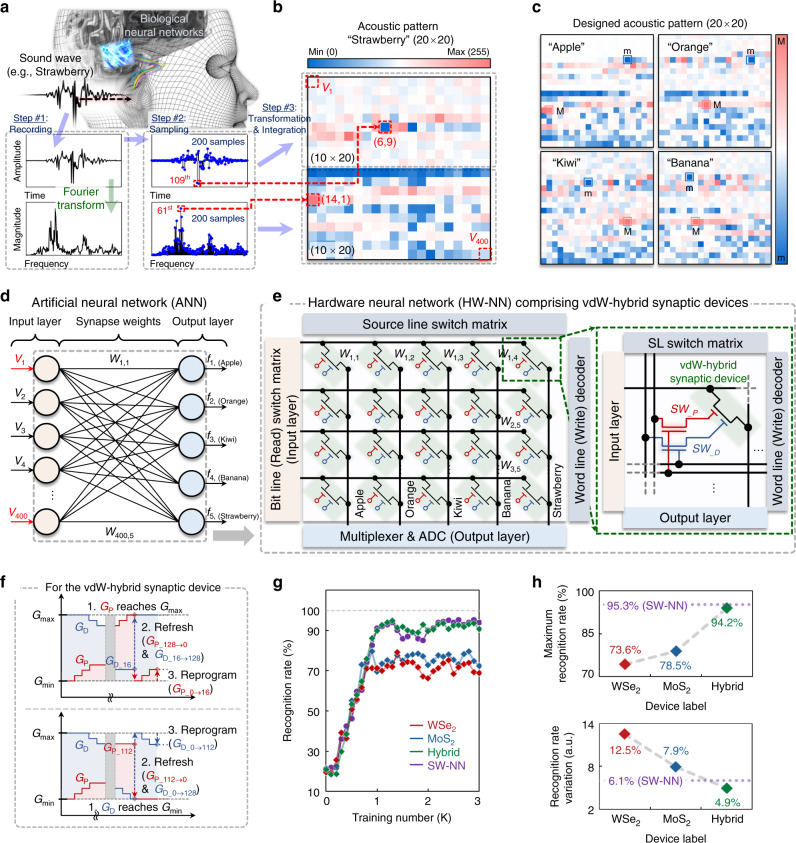
Acoustic pattern recognition task. **a** Design procedure of acoustic pattern (from recording, through transforming, to integrating). **b** Designed 20 × 20 acoustic pattern, where each pixel has a grayscale value in the range between 0 and 255. **c** Designed acoustic pattern. **d** Single-layer artificial neural network (ANN) consisting of input neurons, synapses, and output neurons. **e** The hardware neural network (HW-NN) comprising the vdW-hybrid synaptic device for the conceptual neural networks. **f** Weight update method based on operations of “refresh” and “reprogram” for the vdW-hybrid synaptic device. **g** Acoustic pattern recognition rates based on three ANNs comprising WSe_2_, MoS_2_, or hybrid synaptic devices, and comparison with the rate based on SW-NN. **h** Extracted maximum recognition rates and corresponding variations.

Finally, we demonstrated the feasibility of the vdW-hybrid synaptic device for an HW-NN via a training and inference simulation. For this simulation task, we defined a method to convert vocal signals into acoustic patterns and then prepared training and inference datasets, as shown in Fig. 4a and b. The first step was to express vocal signals as a function of time or frequency. We recorded the sound wave of a spoken word (“strawberry”) and obtained the sound information as a function of time. The sound amplitude vs. time information was transformed to the frequency domain via a Fourier transform. The second step was sampling the sound signals. In this step, we divided the sound signals into 200 time or frequency points. Finally, in the third step, the discrete signal information was transformed into an acoustic image with a 20 × 20 array size, as shown in Fig. 4b. For example, the 109th data point in the sound amplitude vs. time graph and the 61st data point in the sound magnitude vs. frequency graph were transferred into the pixels located at (6,11) and (14,1) in the acoustic pattern, respectively (see dotted red line). Here, each pixel had a grayscale value in the range of 0–255. Datasets with 3000 training and 400 inference acoustic pattern images were prepared similarly for five distinct words: “apple,” “orange,” “kiwi,” “banana,” and “strawberry” (see Fig. 4c). Additional information about the datasets is presented in Supplementary Fig. 23. We also prepared two types of spoken digit datasets consisting of cochleagram patterns or our acoustic patterns, where the Lyon’s auditory model was applied to create the cochleagram patterns (see Supplementary Fig. 24)^47^. Then, as shown in Fig. 4d, we theoretically designed a single-layer artificial neural network (ANN) consisting of 400 input neurons, five output neurons, and 400 × 5 artificial synapses connecting the neurons. The voltage signals (*V*_n_) corresponding to each pixel of the acoustic pattern were assumed to be applied to the input neuron layer. They were multiplied by the synapse weight (*W*_n,m_) and then summed at the output neurons. Consequently, output currents $( {I_{\mathrm{m}} = \mathop {\sum }\nolimits_{n = 1}^{400} W_{{\mathrm{n}},{\mathrm{m}}}V_{\mathrm{n}}} )$ were obtained at the output neuron layer. The synapse weight was defined as the conductance values of the synaptic device (*W* = *G*). Next, the output value (*f*_m_) obtained via the sigmoid activation function $( {f( {I_{\mathrm{m}}}) = \frac{1}{{1 + e^{ - I_{\mathrm{m}}}}}} )$ was compared with each label value (*k*_m_). Finally, the synapse weights were updated via the backpropagation algorithm (details are presented in the METHODS Section). Figure 4e shows the hardware neural network (HW-NN) comprising the vdW-hybrid synaptic devices, which is applicable to the implementation of the conceptual neural networks for acoustic and MNIST digit pattern recognition tasks. Details on this HW-NN are described in Supplementary Fig. 25.

The conductance of the vdW-hybrid synaptic device was updated by only trapping electrons in the WCL, which caused the conductance state to no longer potentiate or depress when *G*_P_ or *G*_D_ reached *G*_max_ or *G*_min_. Therefore, for training the ANN composed of the hybrid devices, we employed a conductance updating method based on the operations of “refresh” and “reprogram” for updating *G*_P_ and *G*_D_. As shown in Fig. 4f (top), when *G*_P_ reached *G*_max_ (*G*_P_128_), both *G*_P_ and *G*_D_ were refreshed to *G*_min_ (*G*_P_128→0_) and *G*_max_ (*G*_D_16→128_). For the operation of “refresh” in terms of implementation in hardware, (i) the peripheral circuits to read the *G*_P_ and *G*_D_ separately and (ii) the physical separation of the channels are required simultaneously. Subsequently, *G*_P_ was reprogrammed to the value of *G*_D_ before the refreshing step (*G*_P_0→16_), maintaining its conductance value (*G* = *G*_P_128_ + *G*_D_16_ = *G*_P_16_ + *G*_D_128_). For the operation of “reprogram” in terms of implementation in hardware, the peripheral circuits and memory are required additionally, which will store the conductance value and write it back to the device. Similarly, as shown in Fig. 4f (bottom), when *G*_D_ reached *G*_min_ (*G*_D_0_), *G*_P_ and *G*_D_ were refreshed, and then *G*_D_ was reprogrammed to the value of *G*_P_ before the refreshing step (*G*_D_128→112_). We also employed a conductance updating method without the operations of “refresh” and “reprogram” as shown in Supplementary Fig. 26. The training process was conducted for three types of ANNs composed of hybrid (green curve), WSe_2_ (red curve), and MoS_2_ (blue curve) devices, and we calculated the recognition rate every 100 training steps, as shown in Fig. 4g. The same training process was performed with an SW-NN (purple curve), for which the synaptic weights were updated using the Widrow–Hoff learning rule^48^. Also, the recognition rates for the acoustic patterns formed with frequency- and/or time-domain data are provided in Supplementary Fig. 27. After the training and inference tasks, as shown in Fig. 4h, the maximum recognition rates and corresponding variation values, which denote the degree of fluctuating in learning curves, were examined. The maximum recognition rate/variation values were 73.6%/12.5%, 78.5%/7.9%, and 94.2%/4.9% for the WSe_2_, MoS_2_, and vdW-hybrid synaptic devices. The values for the hybrid device were closest to those for the SW-NN (95.3%/6.1%). Similar training and inference analyses were performed for (i) various spike conditions (number, amplitude, duration, and frequency of spikes) with the designed acoustic patterns, (ii) different layer numbers of the ANN (single- and multi-layer) using the MNIST datasets, and (iii) the two types of spoken digit datasets consisting of cochleagram patterns or acoustic patterns, as shown in Supplementary Figs. 28, 29, 30, and Supplementary Table 2, respectively^21,49^.

## Discussion

We developed a vdW-hybrid synaptic device featuring linear and symmetric update characteristics by utilizing WSe_2_ and MoS_2_ hybrid channels that are specialized for linear conductance potentiation and depression, respectively. Excellent conductance controllability of the vdW-hybrid synapse was achieved by utilizing only electron-trapping phenomenon in the WCL. The vdW-hybrid synaptic device exhibited nonlinearity and symmetricity of 1.9/1.9 (β_P_/β_D_) and 13.26, respectively, an effective conductance-state ratio of 85.94% for threshold_Δ*G*_ = 0.3%, a very small variation (~1%) after state changes by excitatory and inhibitory spikes, a CCV of <1%, and an RSD of 0.05/0.03 (weight potentiation/depression). Such synaptic characteristics are highlighted in Supplementary Table 1, where our synaptic device is investigated and compared with other devices reported heretofore. Through in-depth analysis and characterization of the vdW-hybrid synaptic device, we demonstrated the feasibility of the device for an HW-NN. It exhibited high recognition rates close to those for an SW-NN via training and inference simulation, in which our designed acoustic patterns were employed. Using this hybrid synaptic device, we achieved recognition of 93.8% for an acoustic pattern recognition task, which was close to that for the SW-NN (95.3%). This work indicates the potential for building HW-NNs for highly accurate brain-inspired computing.

## Methods

### Fabrication of the synaptic devices

The individual electrodes for the WCT with a width of 20 μm were patterned on a 90-nm-thick SiO_2_ oxide layer on a heavily B-doped Si substrate using an optical lithography process, followed by the deposition of 10-nm-thick Ti and 30-nm-thick Au using an electron-beam evaporator. *h*-BN flakes were mechanically transferred onto the WCTs via a residue-free transfer method based on adhesion energy engineering^12^. Then, CF_4_ plasma treatment was conducted on the *h*-BN flakes using a plasma machine (Miniplasma Cube, PLASMART). For stabilizing the chamber conditions, CF_4_ gas flowed for 1 min before the CF_4_ plasma treatment. The CF_4_ plasma treatment conditions were as follows: reactive ion etcher powers of 5, 10, and 20 W; a plasma pressure of 500 mTorr; a CF_4_ flow rate of 5 sccm; and treatment times of 10, 20, and 90 s. The WSe_2_ and MoS_2_ flakes were then transferred onto the WCL/*h*-BN via the same transfer method. The postsynaptic and presynaptic electrodes (distance between the two electrodes and width of the electrodes were 5 μm) were patterned on the WSe_2_/WCL/*h*-BN (potentiation channel) and MoS_2_/WCL/*h*-BN (depression channel) structure, followed by 10-nm-thick Pt contact for the potentiation channel and Ti contact for the depression channel and 50-nm-thick Au pad deposition.

### Characterization of the synaptic devices

For structural and elemental analyses of the WSe_2_/WCL/*h*-BN and MoS_2_/WCL/*h*-BN regions, X-TEM (JEM ARM 200 F) and EELS (GIF Quantum ER, 200 keV) measurements were performed. Raman analysis was performed at various positions on the WSe_2_/WCL/*h*-BN and MoS_2_/WCL/*h*-BN samples using a WITec micro-Raman spectrometer system with a frequency-doubled Nd-doped yttrium aluminum garnet (Nd-YAG) laser beam (532-nm laser excitation). AFM was performed using an NX10 system (Park Systems Corp.). Electrical measurements of the synaptic devices were performed using an HP-4155A semiconductor parameter analyzer connected to a voltage spike generator (Keysight, 33500B). The aforementioned measurement setup for the synaptic devices is described in detail in Supplementary Fig. 2.

### Weight update for synaptic devices

Currents at output neurons were transformed by a sigmoid activation function, resulting in output neuron signals (*f*). Based on the delta value (*δ*), which is difference between the output neuron signals and the label values (*k*) for input patterns (*δ* = *k* − *f*), the synaptic weight was determined to be potentiated or depressed. If *δ* > 0 (potentiation phase), then *G* is increased. In the depression phase (*δ* < 0), *G* is decreased. These conductance changes (∆*G*) were determined by the following equations:

$$G_{{\mathrm{n}} + 1} = G_{\mathrm{n}} + {\mathrm{{\Delta}}}G_{\mathrm{P}} = G_{\mathrm{n}} + {\upalpha}_{\mathrm{P}}e^{ - \beta _{\mathrm{P}}\frac{{G_{\mathrm{n}} - G_{{\mathrm{min}}}}}{{G_{{\mathrm{max}}} - G_{{\mathrm{min}}}}}}\,\left( {{\Delta}G \,> \, 0, G \uparrow } \right),$$

$$G_{{\mathrm{n}} + 1} = G_{\mathrm{n}} + {\mathrm{{\Delta}}}G_{\mathrm{D}} = G_{\mathrm{n}} - {\upalpha}_{\mathrm{D}}e^{ - \beta _{\mathrm{D}}\frac{{G_{{\mathrm{max}}} - G_{\mathrm{n}}}}{{G_{{\mathrm{max}}} - G_{{\mathrm{min}}}}}}\,\left( {{\Delta}G \, < \, 0,\,G \downarrow } \right).$$

In these equations, *G*_n+1_ and *G*_n_ denote the synaptic conductance when the n + 1th and nth pulses are applied, and parameters α and β are the conductance change amount and the nonlinearity, respectively. Fitting results are provided in Supplementary Table 3. The above pattern recognition processing was implemented with MATLAB.

## Supplementary information

Supplementary Information

## Data Availability

The data that support the findings of this study are available from the corresponding author upon request.

## Code availability

Code from this study (MATLAB scripts) is available from the corresponding author upon request.
