# Supplementary material for: Artificial van der Waals hybrid synapse and its application to acoustic pattern recognition
Source: Nat Commun. 2020 Aug 7;11:3936. doi: 10.1038/s41467-020-17849-3 (PMC7414205; doi:10.1038/s41467-020-17849-3)
Supplement: Supplementary file 1 — Supplementary Information [file 41467_2020_17849_MOESM1_ESM.pdf]

Supplementary Information:

Artificial Van der Waals Hybrid Synapse and its Application to Acoustic Pattern Recognition

Seunghwan Seo,[1] Beom-Seok Kang,[1] Je-Jun Lee,[1] Hyo-Jun Ryu,[1,2] Sungjun Kim,[1,3] Hyeongjun Kim,[1] Seyong Oh,[1] Jaewoo Shim,[4] Keun Heo,[1] Saeroonter Oh,[5] Jin-Hong Park[1,6,*]

[1] *Department of Electrical and Computer Engineering, Sungkyunkwan University, Suwon 16419, Korea*

[2] *Semiconductor R&D Center, Samsung Electronics Co. Ltd., Hwasung 18448, Korea*

[3] *Foundry Division, Samsung Electronics Co. Ltd., Youngin 17113, Korea*

[4] *Department of Mechanical Engineering, Massachusetts Institute of Technology (MIT), Cambridge, MA 02139, USA*

[5] *Division of Electrical Engineering, Hanyang University, Ansan 15588, Korea*

[6] *Sungkyunkwan University Advanced Institute of Nanotechnology, Sungkyunkwan University, Suwon 16417, Korea*

* Corresponding author's email address: jhpark9@skku.edu (J.-H. P).

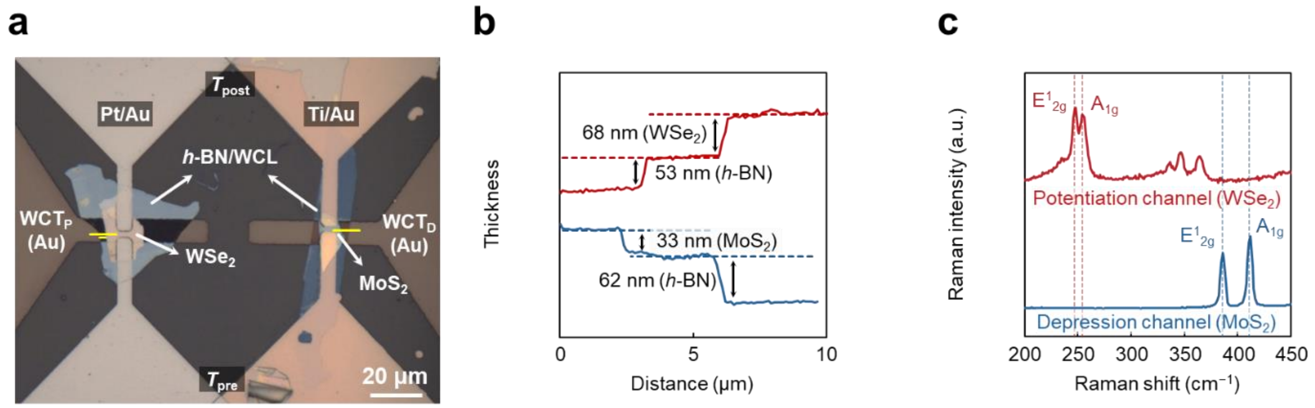

Supplementary Figure 1 | Characterization of the vdW-hybrid synaptic device. a, Optical image of the vdW-hybrid synaptic device. **b,** Thickness profiles on WSe_2 (53 nm)/*h*-BN (68 nm) and MoS_2 (33 nm) *h*-BN (62 nm) structures (yellow line in Supplementary Fig. 1a), which were confirmed via atomic force microscopy. **c,** Raman spectra for the potentiation-channel (WSe_2) and depression-channel regions (MoS_2). For the WSe_2 flake, two conventional Raman peaks (E^1_{2g} and A_{1g}) were observed near 245 and 255 cm^{-1}, corresponding to the in- and out-of-plane vibrations for bulk WSe_2, respectively.[1] For the MoS_2 flake, two conventional Raman peaks (E^1_{2g} and A_{1g}) were observed near 384 and 409 cm^{-1}.

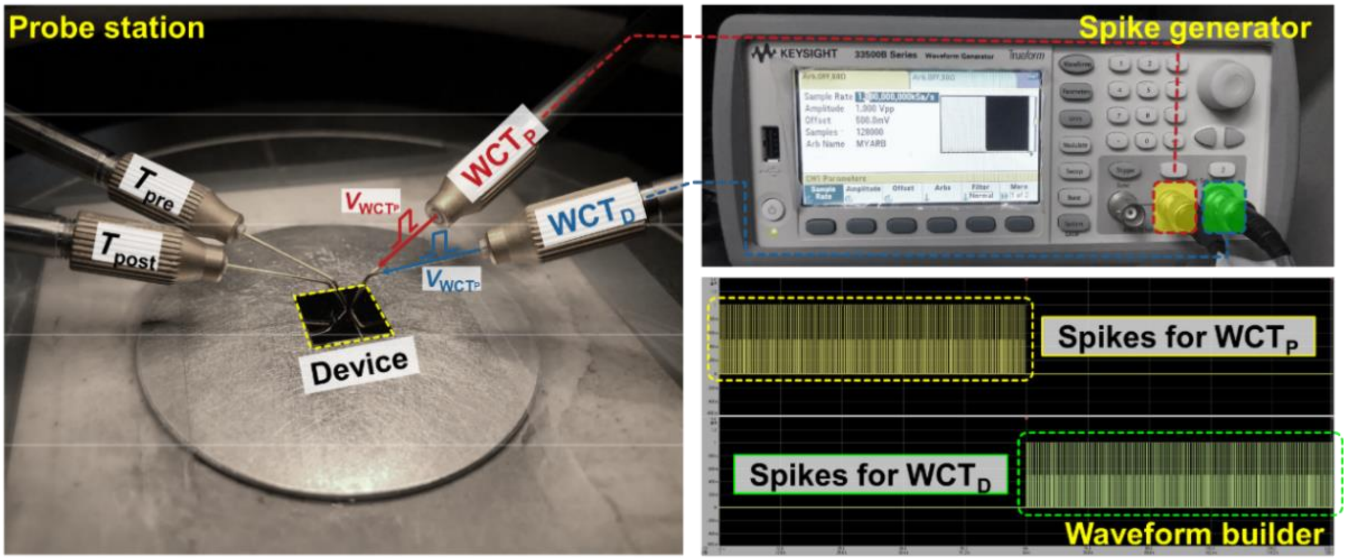

Supplementary Figure 2 | Measurement setup for the vdW-hybrid synaptic device.

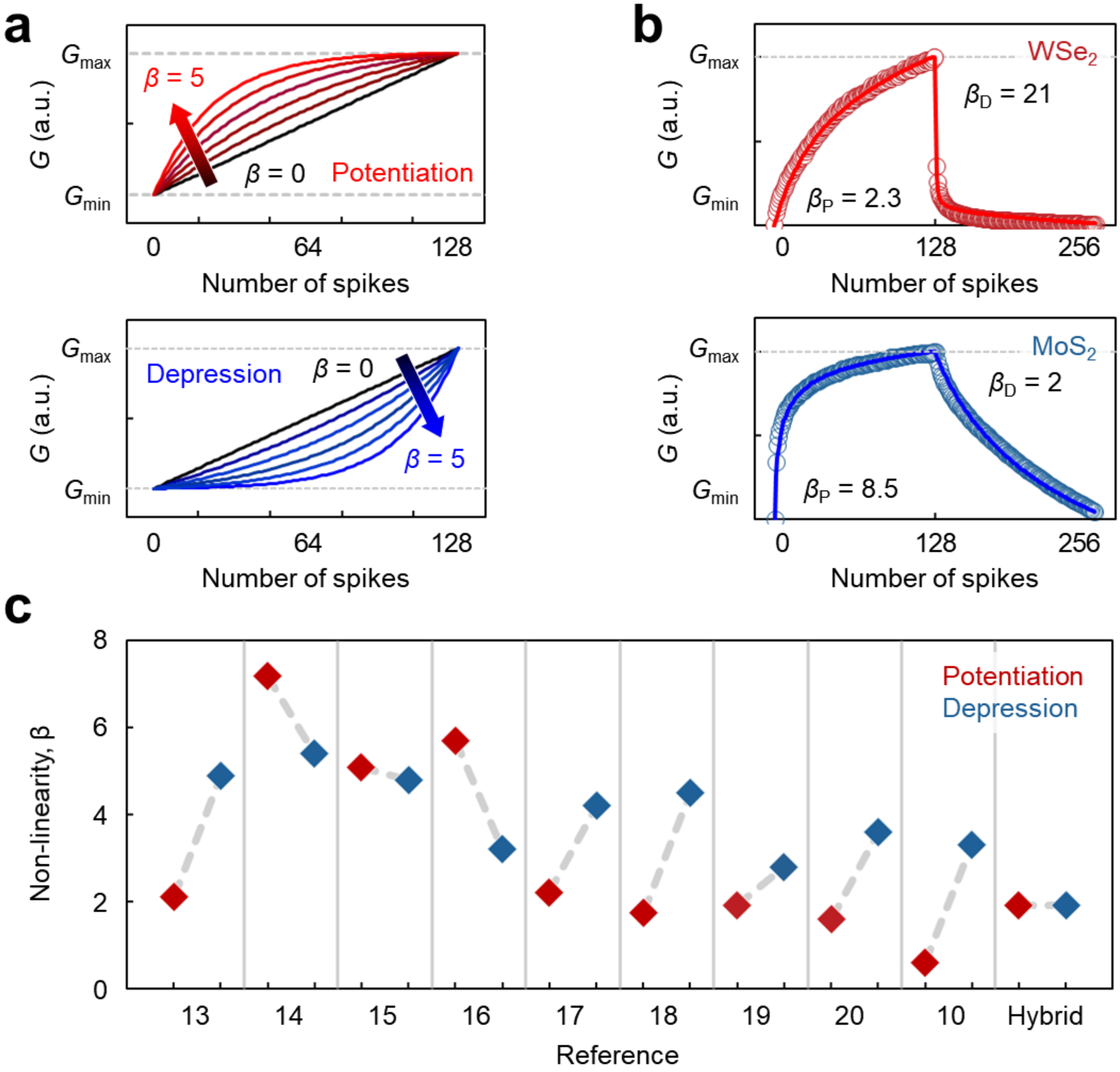

Supplementary Figure 3 | Nonlinearity analysis of the LTP/LTD curves. a, LTP and LTD curves with respect to nonlinearity (β) ranging from 0 to 5. **b,** LTP and LTD curve fitting, $\beta_{P/D}$ = 2.3/21 (WSe_2) and = 8.5/2 (MoS_2). **c,** Nonlinearity comparison of various synaptic devices reported heretofore and this hybrid device. See also Supplementary Note 1.

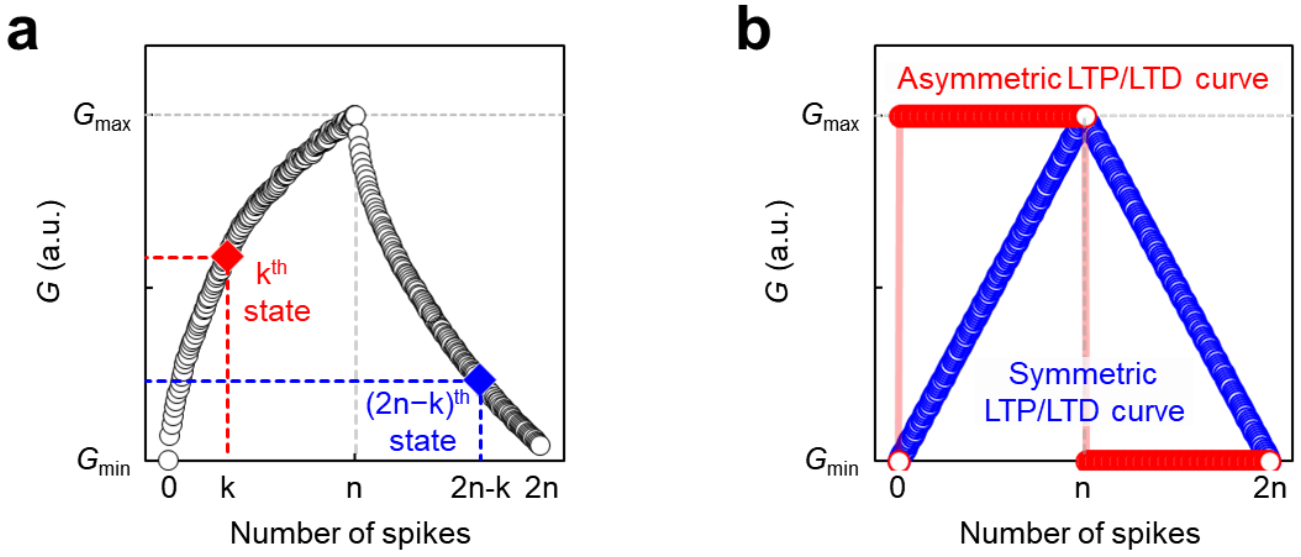

Supplementary Figure 4 | Symmetricity analysis of the LTP/LTD curves. a, LTP/LTD curves with 2n conductance states and the kth (red dot) and $(2n\text{-}k)^{th}$ (blue dot) conductance states. **b,** Asymmetric (symmetric error = ∞) and symmetric (symmetric error = 0) LTP/LTD curves. See also Supplementary Note 2.

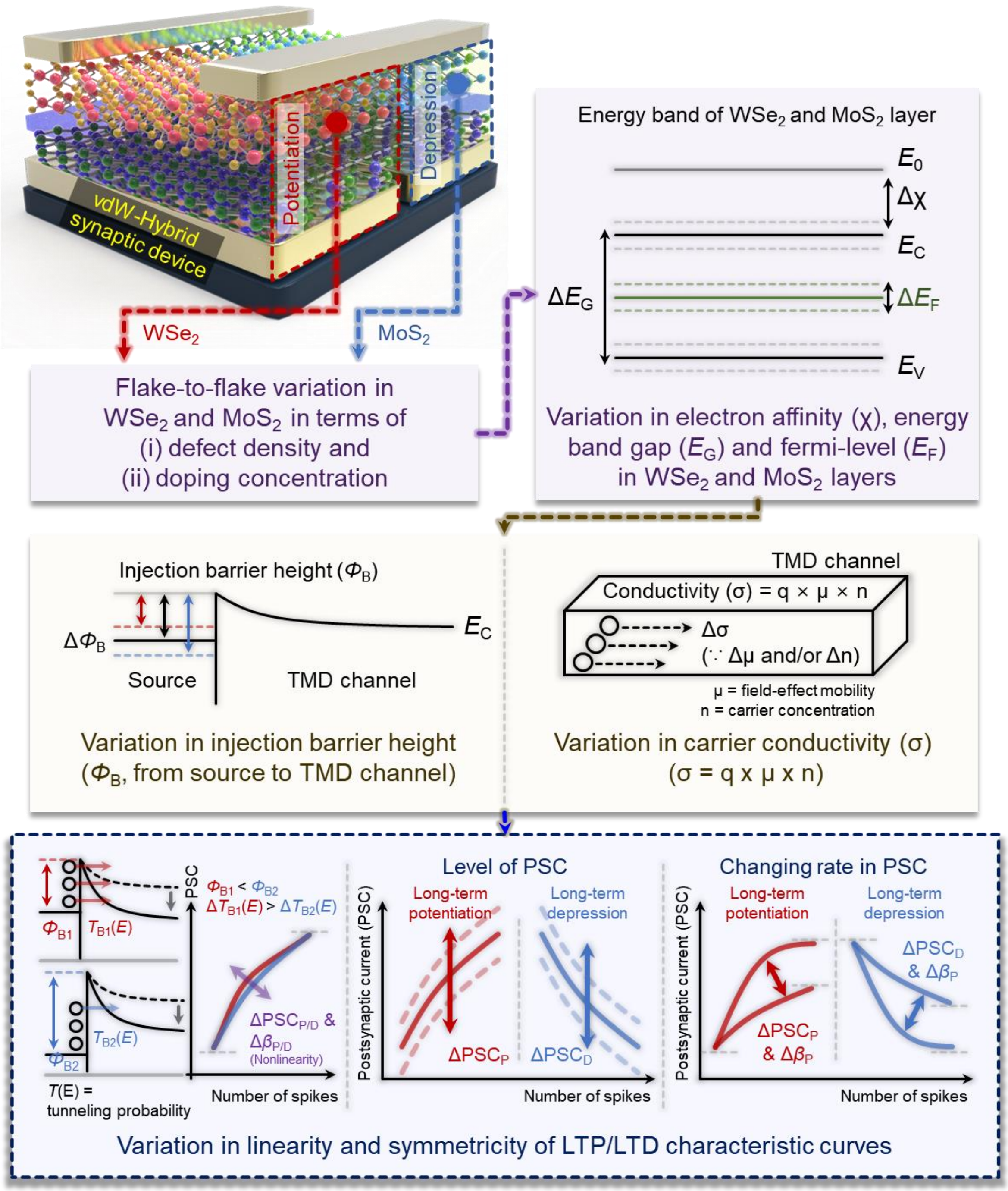

Supplementary Figure 5 | Variation in the linearity and symmetricity of the LTP and LTD curves with respect to the variation in WSe_2 and MoS_2 layers in terms of defect density and doping concentration. For the WSe_2 and MoS_2 layers, there are flake-to-flake variations in the vdW channel in terms of defect density and doping concentration, which consequently affect (i) the injection barrier height (from source to channel) and (ii) the carrier conductivity (through channel) by changing the energy band properties of 2D vdW flakes. Here, the conductivity is determined by a field-effect mobility and a doping concentration. Therefore, such variations will influence the synapse characteristics including the linearity and symmetricity of the LTP and LTD curves. This is because conductance levels and their changing rate in the LTP/LTD curves are strongly dependent on the injection barrier height and the channel conductivity.

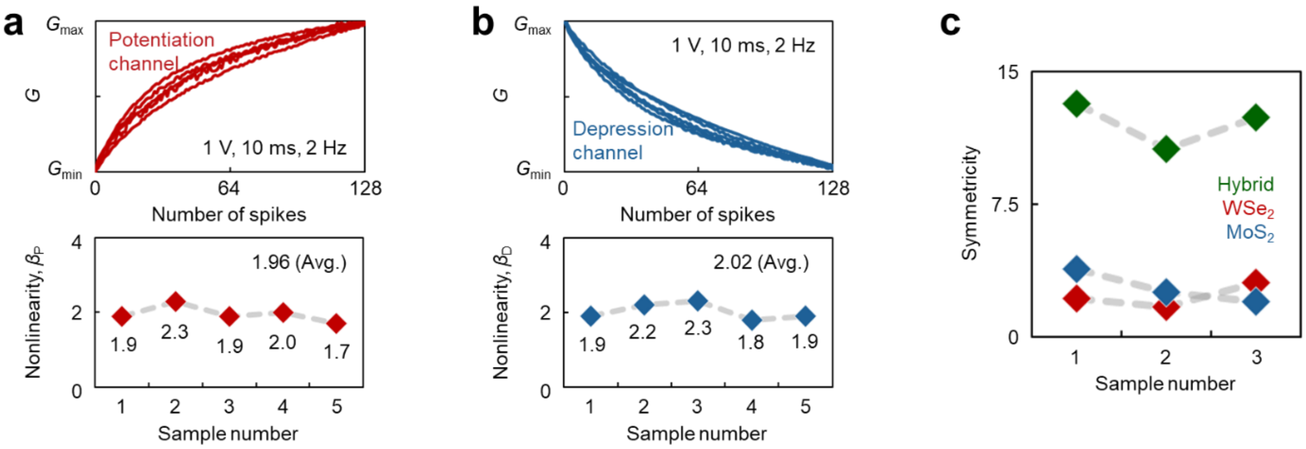

Supplementary Figure 6 | Spread of the nonlinearity obtained in multiple devices. a, Long-term potentiation (LTP) curves for the potentiation channel in five devices (top) and their nonlinearity (bottom). **b,** Long-term depression (LTD) curves for the depression channel in five devices (top) and their nonlinearity (bottom). **c,** The spread of the symmetricity obtained in three devices.

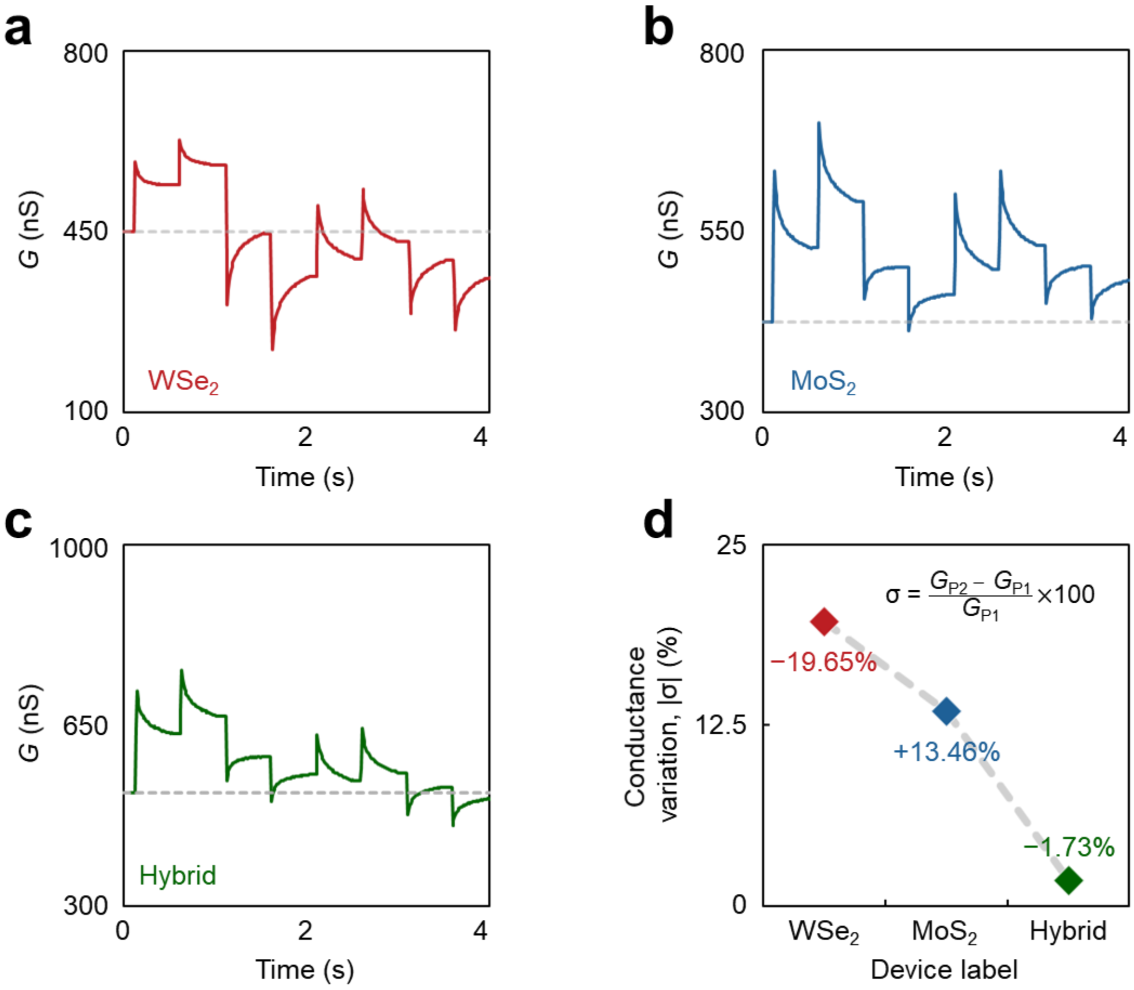

Supplementary Figure 7 | Conductance variations for the three types of synaptic devices. a,b,c, Spike responses for the WSe_2 synaptic device (**a**), MoS_2 synaptic device (**b**), and vdW-hybrid synaptic device (**c**). **d,** Conductance variation with respect to the type of device. See also Supplementary Note 3.

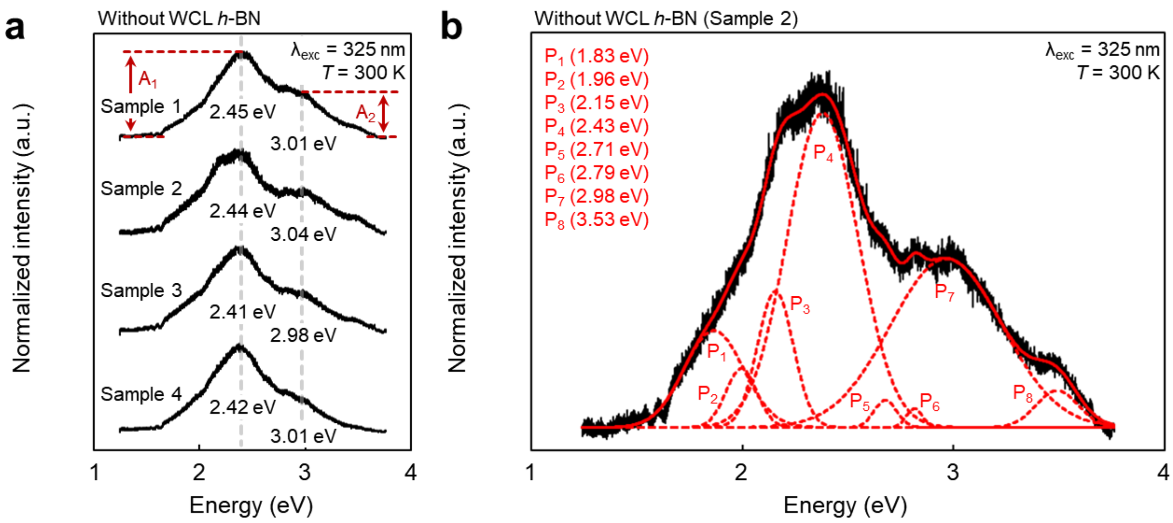

Supplementary Figure 8 | Photoluminescence (PL) analysis for *h*-BN samples without WCL. a, PL spectra measured on four different *h*-BN samples without WCL. **b,** Decomposition of the PL spectrum (Sample 2). See also Supplementary Note 4.

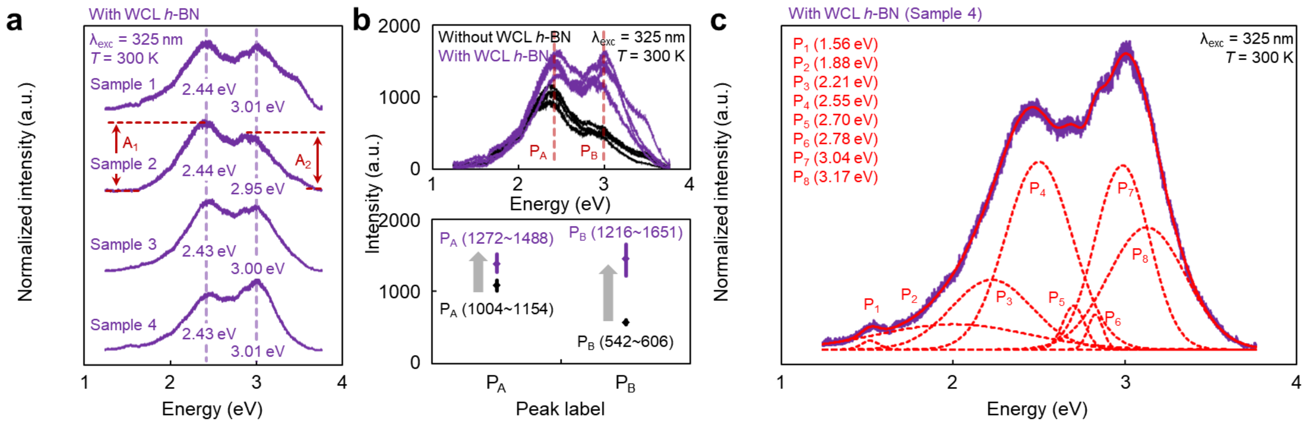

Supplementary Figure 9 | Photoluminescence (PL) analysis for *h*-BN samples with WCL. a, PL spectra measured on four different *h*-BN samples with WCL. **b,** Comparison of PL spectra measured on the *h*-BN samples without/with WCL. **c,** Decomposition of the PL spectrum (Sample 4). See also Supplementary Note 4.

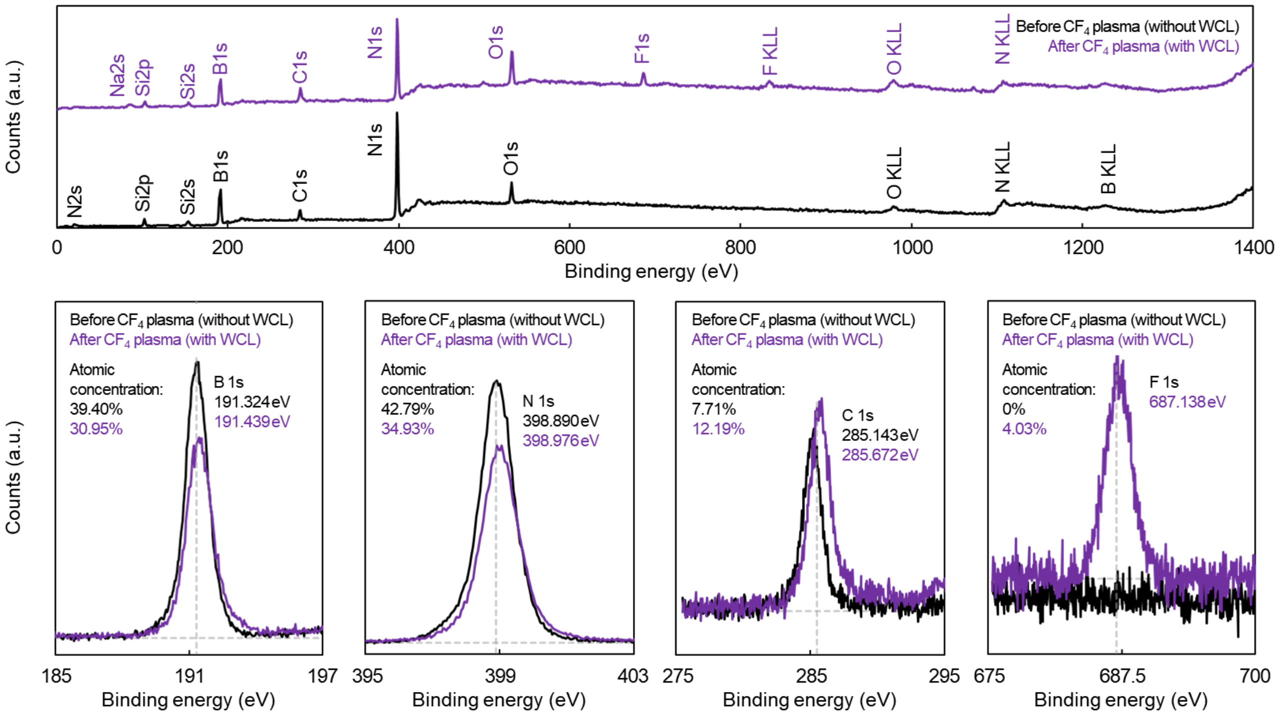

Supplementary Figure 10 | Micro-XPS analysis for *h*-BN layers without/with WCL. See also Supplementary Note 4.

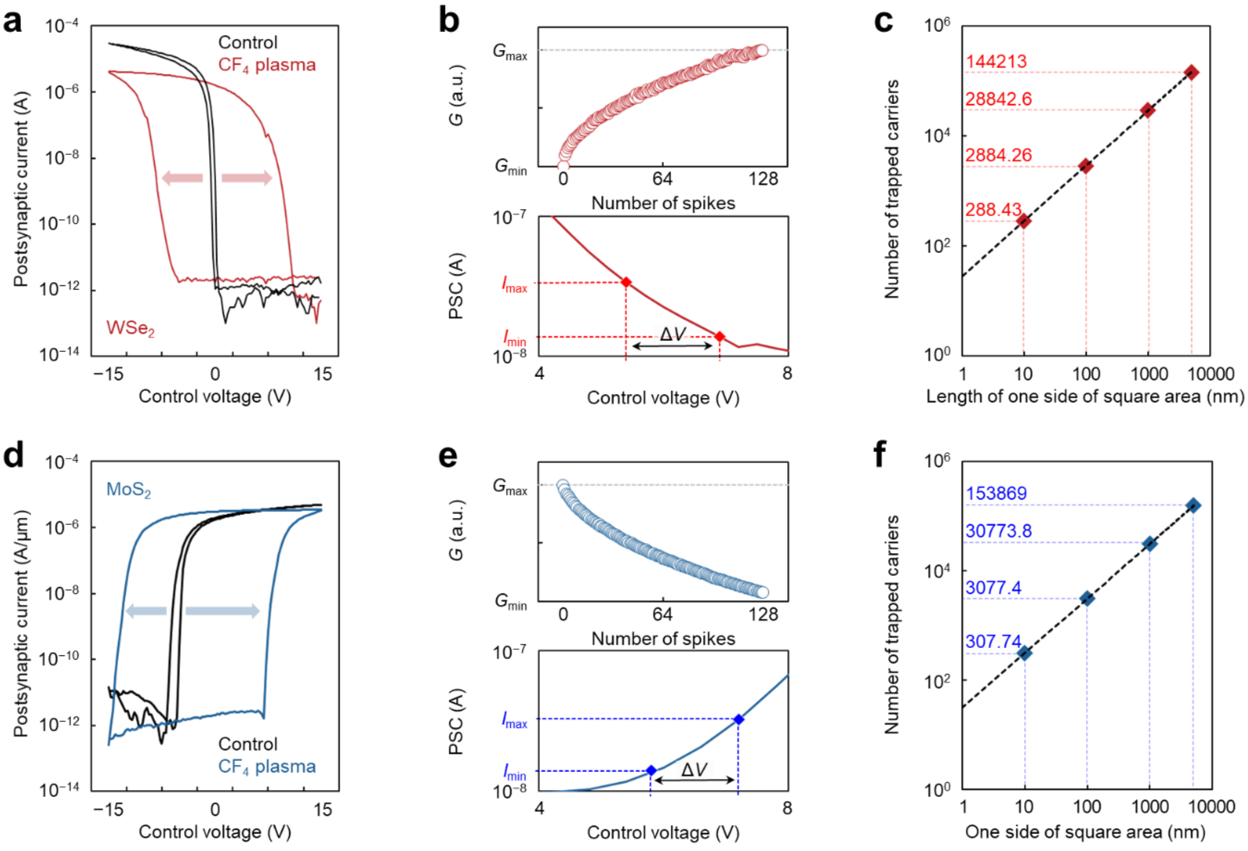

Supplementary Figure 11 | Electrical transfer curves of the potentiation and depression channels and their approximate uniform trap density in the WCL. a,d, Electrical transfer curves of the potentiation (**a**) and depression (**d**) channels without/with the WCL formed by CF_4 plasma treatment. **b,e,** Control voltage range between G_{min} and G_{max}. **c,f,** Number of trapped carriers with respect to the length of one side of the square area. See also Supplementary Note 5.

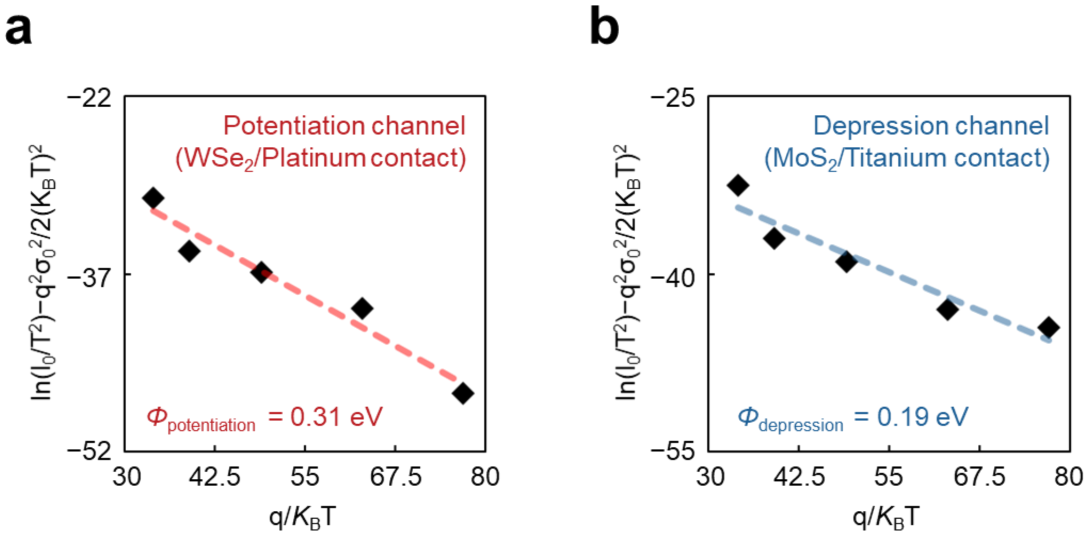

Supplementary Figure 12 | Extraction of the injection barrier height in the potentiation and depression channel. a, Extracted hole injection barrier height in the potentiation channel. **b,** Extracted electron injection barrier height in the depression channel. See also Supplementary Note 6.

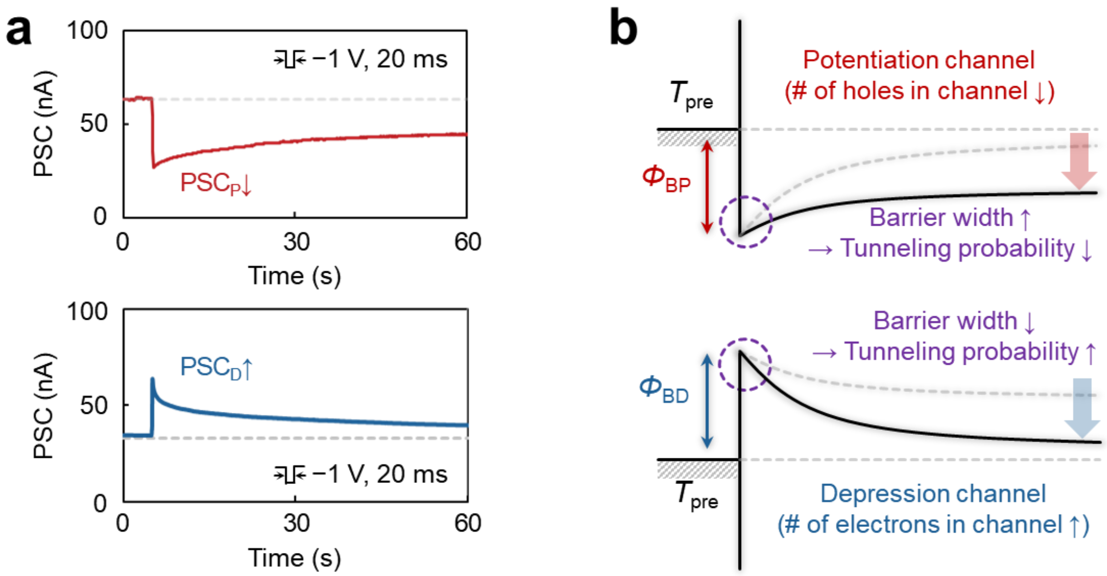

Supplementary Figure 13 | PSC response to a negative voltage spike in the potentiation and depression channels. a, Inhibitory PSC (IPSC) and exhibitory PSC (EPSC) curves in the potentiation and depression channels. **b,** Energy band alignment when an inhibitory spike was applied. See also Supplementary Note 7.

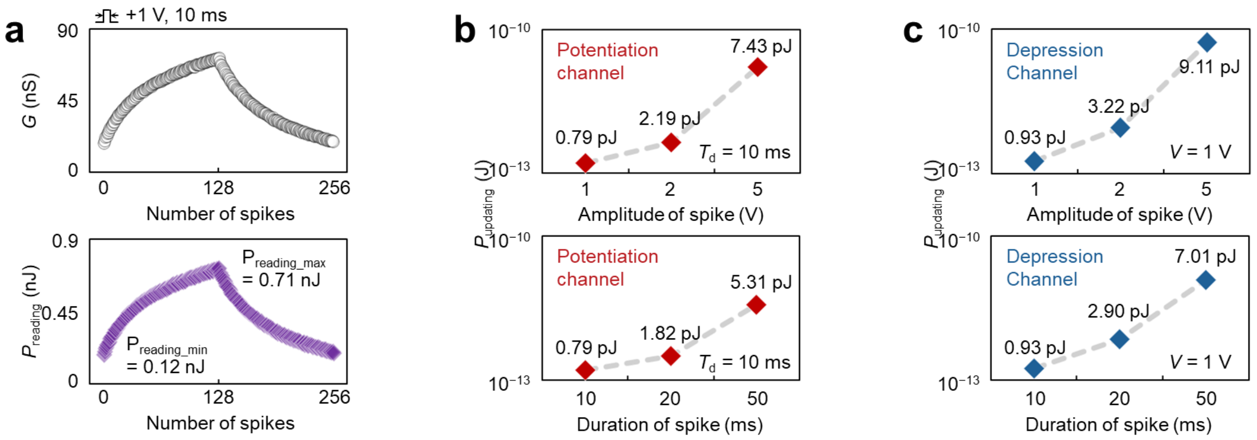

Supplementary Figure 14 | The operating (reading and updating) energy of the vdW-hybrid synaptic device. a, Reading energy of the vdW-hybrid synaptic device. **b,** Updating energy of potentiation channel with respect to the amplitude and duration of spike. **c,** Updating energy of depression channel with respect to the amplitude and duration of spike. See also Supplementary Note 10.

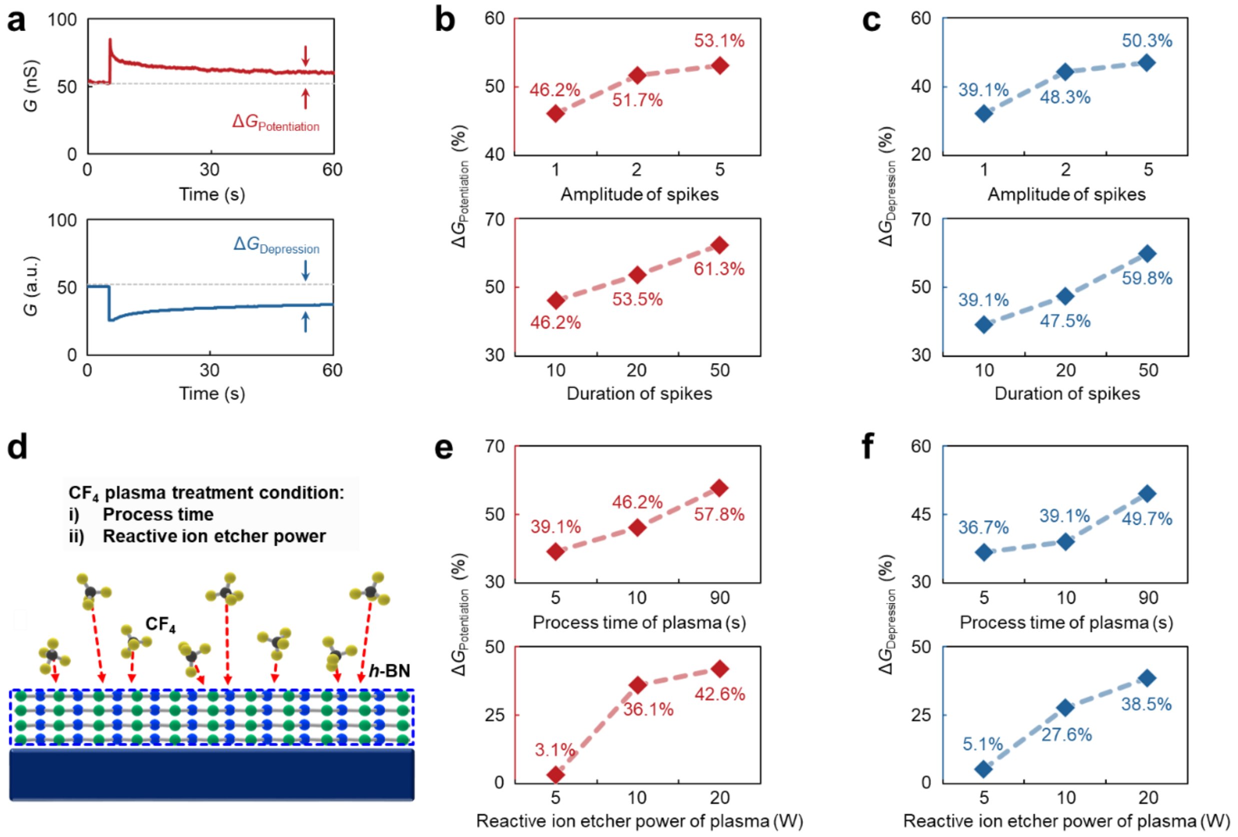

Supplementary Figure 15 | PSC response with respect to the conditions of the spikes and CF_4 plasma treatment. a, EPSC and IPSC curves for the potentiation and depression channels exhibiting $\Delta G_{\mathrm{Potentiation}}$ and $\Delta G_{\mathrm{Depression}}$. **b,c,** $\Delta G_{\mathrm{Potentiation}}$ (**b**) and $\Delta G_{\mathrm{Depression}}$ (**c**) with respect to the amplitude (1–5 V) and duration (10–50 ms) of the spikes. **d,** Illustration of the CF_4 plasma treatment on *h*-BN. **e,f,** $\Delta G_{\mathrm{Potentiation}}$ (**e**) and $\Delta G_{\mathrm{Depression}}$ (**f**) with respect to the plasma process time (5–90 s) and reactive ion etcher power in the plasma process (5–20 W). See also Supplementary Note 11.

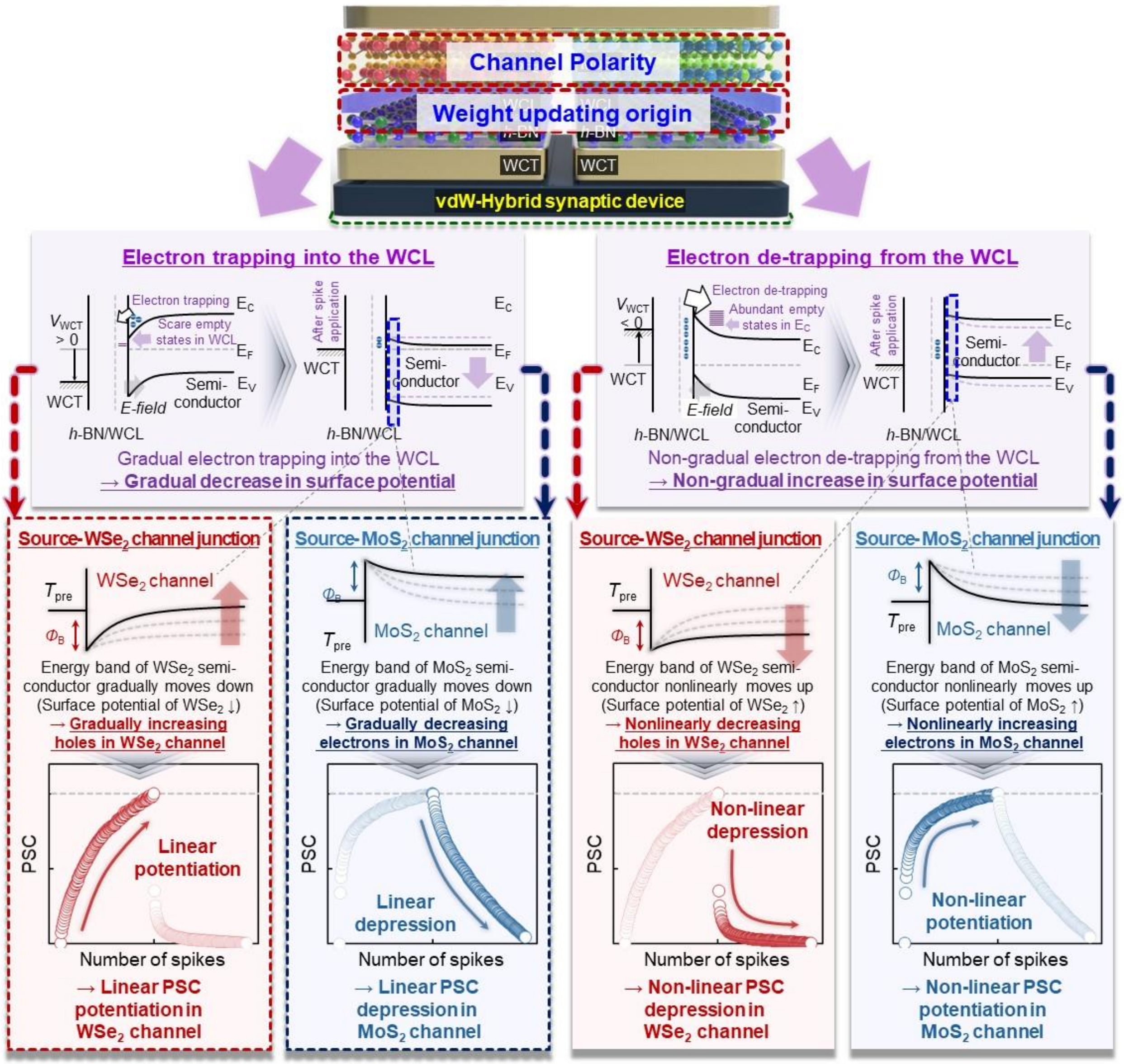

Supplementary Figure 16 | Linearity of PSC determined by the weight updating origin and the channel polarity. See also Supplementary Note 9.

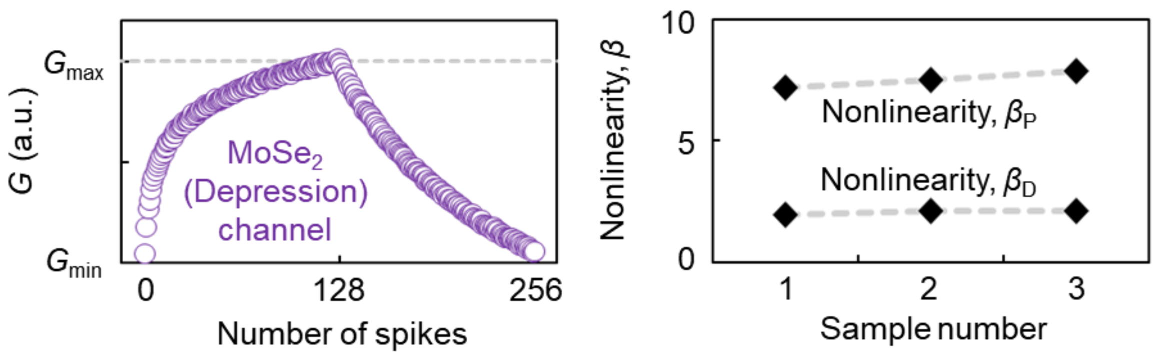

Supplementary Figure 17 | LTP / LTD curves and nonlinearity values extracted from three $MoSe_2$ channel synapse devices.

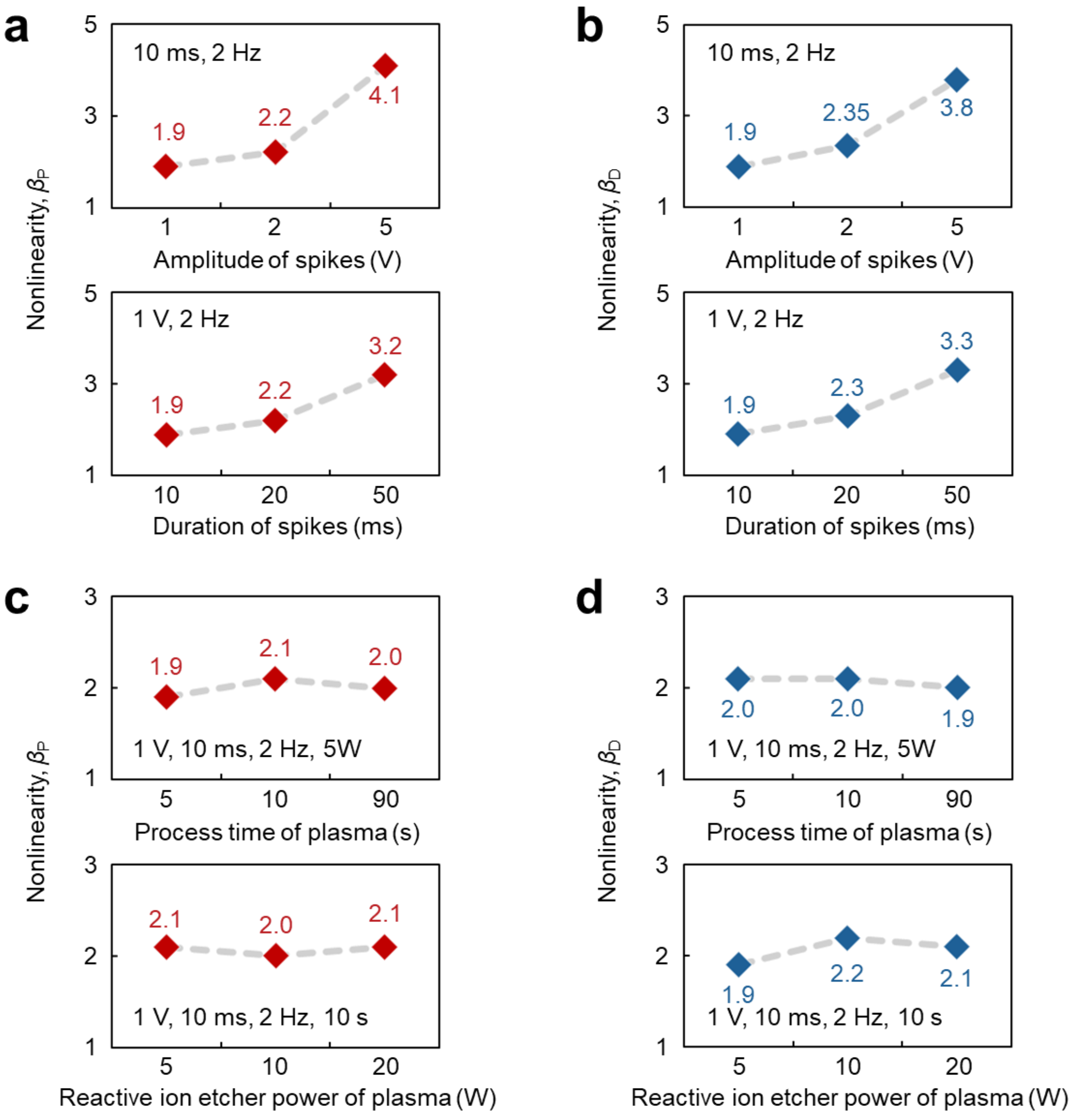

Supplementary Figure 18 | Nonlinearity with respect to the conditions of spikes and CF_4 plasma treatment. a,b, Nonlinearity in the potentiation channel (**a**) and depression channel (**b**) with respect to the amplitudes (1-5 V) and durations (10-50 ms) of the spikes. **c,d,** Nonlinearity in the potentiation channel (**c**) and depression channel (**d**) with respect to the process time (5-90 s) and reactive ion etcher power (5-20 W) of the CF_4 plasma treatment. See also Supplementary Note 12.

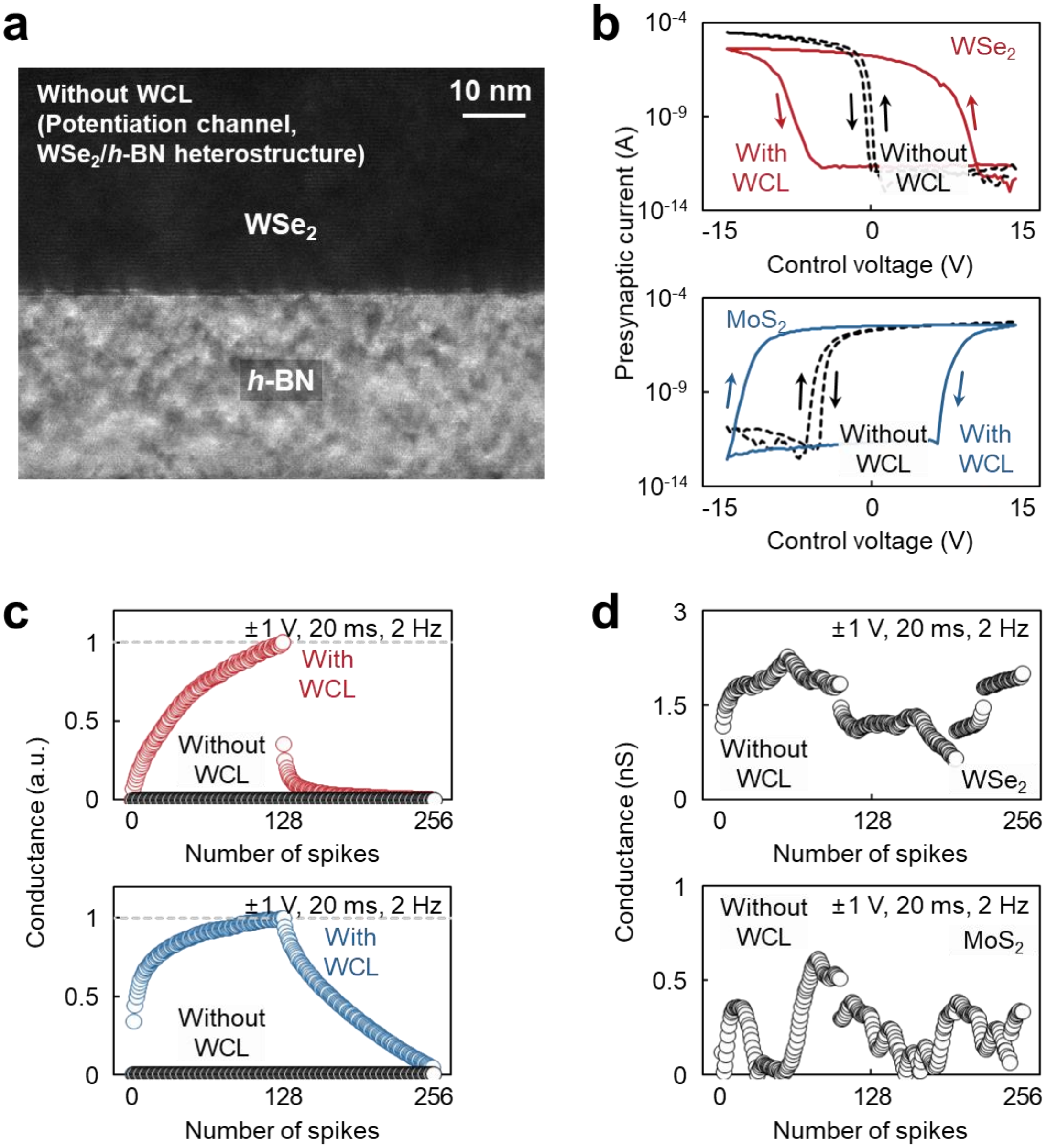

Supplementary Figure 19 | Spike response in potentiation and depression channel without WCL. a, X-TEM images of the potentiation channel (WSe_2/*h*-BN heterostructure). **b,** Electrical transfer curves of the potentiation (WSe_2, top) and depression (MoS_2, bottom) channels without/with the WCL formed by CF_4 plasma treatment. **c,** Long-term potentiation and depression curves of the potentiation (top) and depression (bottom) channels without/with the WCL. **d,** Spike responses of the potentiation (top) and depression (bottom) channels without the WCL.

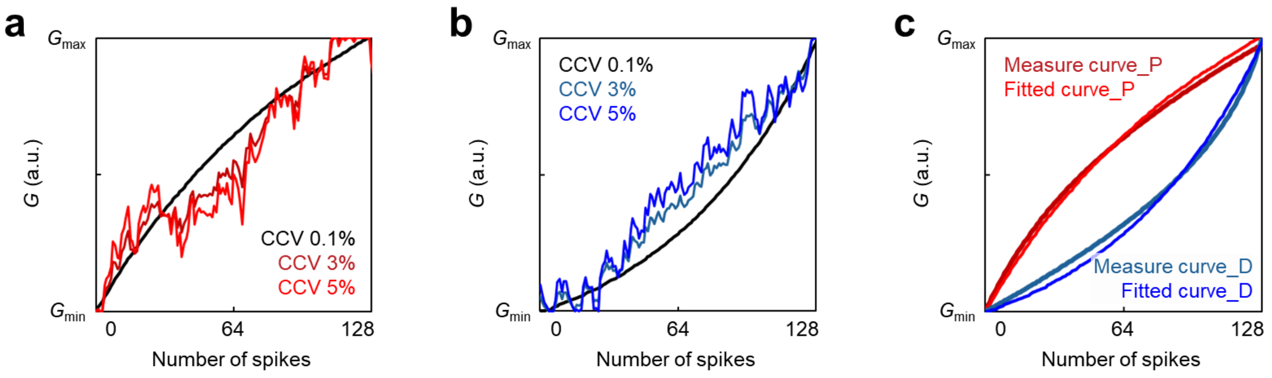

Supplementary Figure 20 | Cycle-to-cycle variation (CCV) of the synaptic devices. a,b, Potentiation (**a**) and depression (**b**) curves for different CCVs (0.1%, 3%, and 5%). **c,** Potentiation/depression curves of the hybrid device and the fitted potentiation/depression curves with 0.1% CCV. See also Supplementary Note 13.

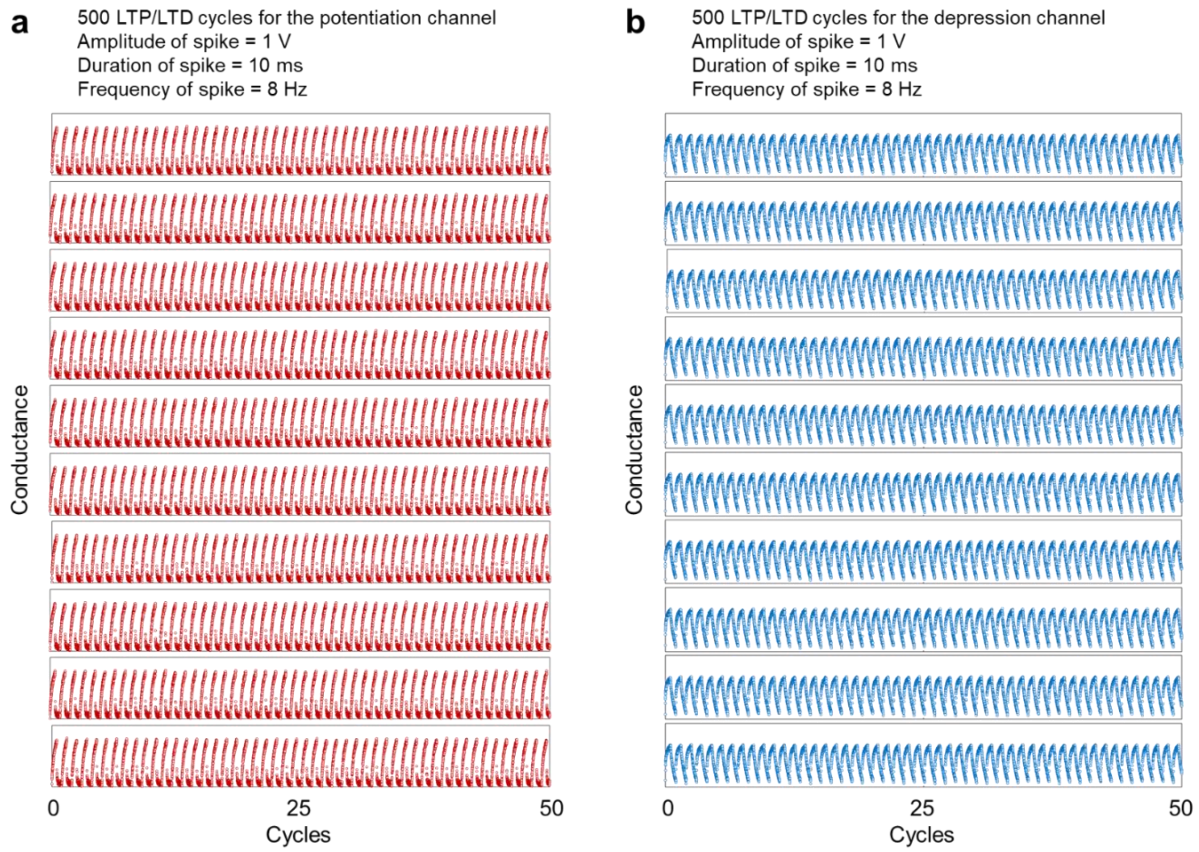

Supplementary Figure 21 | Endurance of the vdW-hybrid synaptic device. a,b, 500 LTP/LTD cycles (128,000 ($10>^5$) weight updating) for the potentiation channel (**a**) and depression channel (**b**) of the vdW-hybrid device.

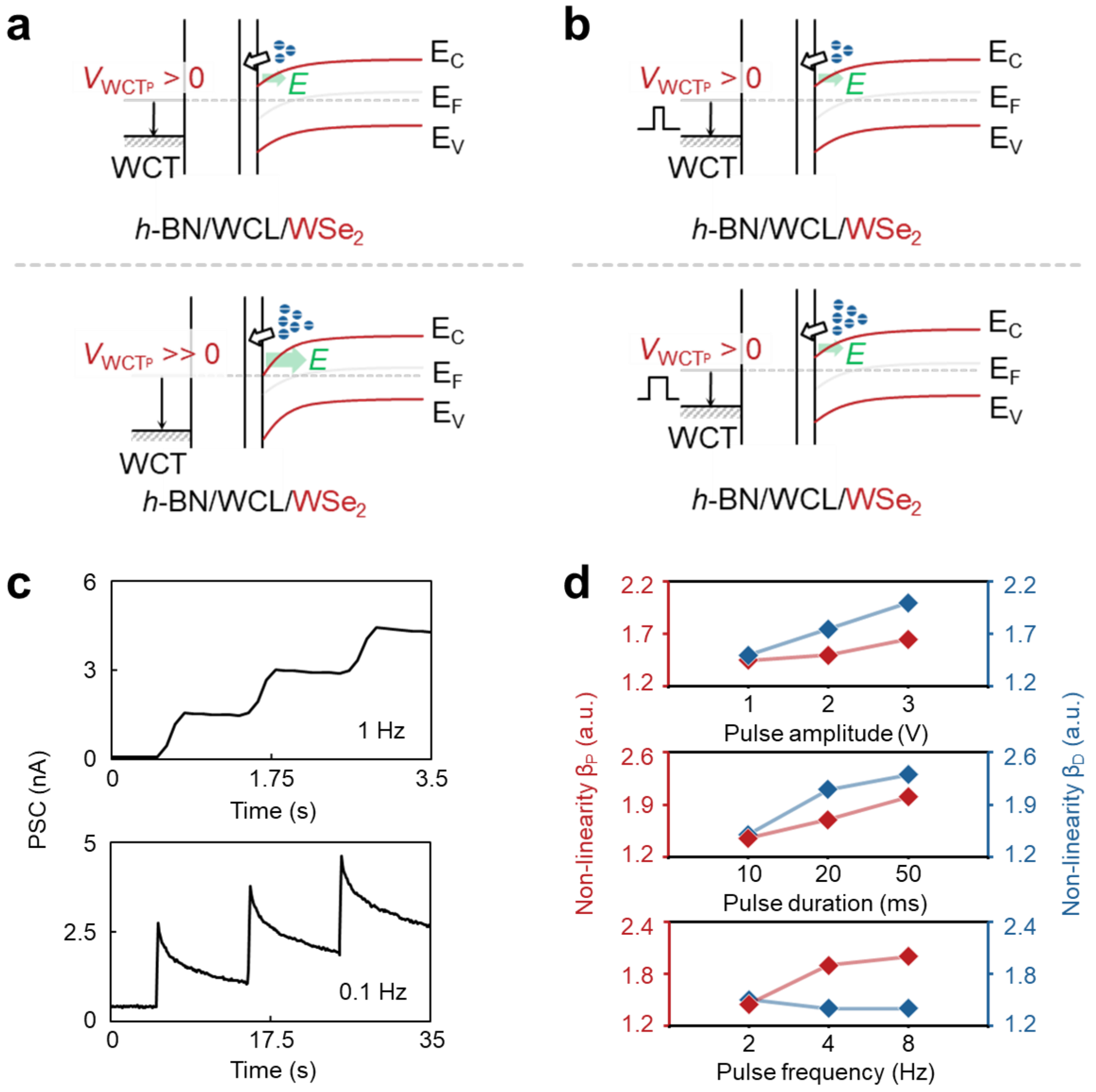

Supplementary Figure 22 | Dependence of dynamic range and symmetricity on spike conditions. a, Energy band alignment when a weak (top) or strong (bottom) exhibitory spike was applied. **b,** Energy band alignment when a short (top) or long (bottom) exhibitory spike was applied. **c,** Spike response of PSC, where spikes with high (1 Hz) and low (0.1 Hz) frequency were applied to the WCT. **d,** Extracted nonlinearity for the potentiation and depression channels with respect to the amplitude, duration, and frequency of spikes, where 128 exhibitory and inhibitory spikes were applied in a row to the WCT. See also Supplementary Note 14.

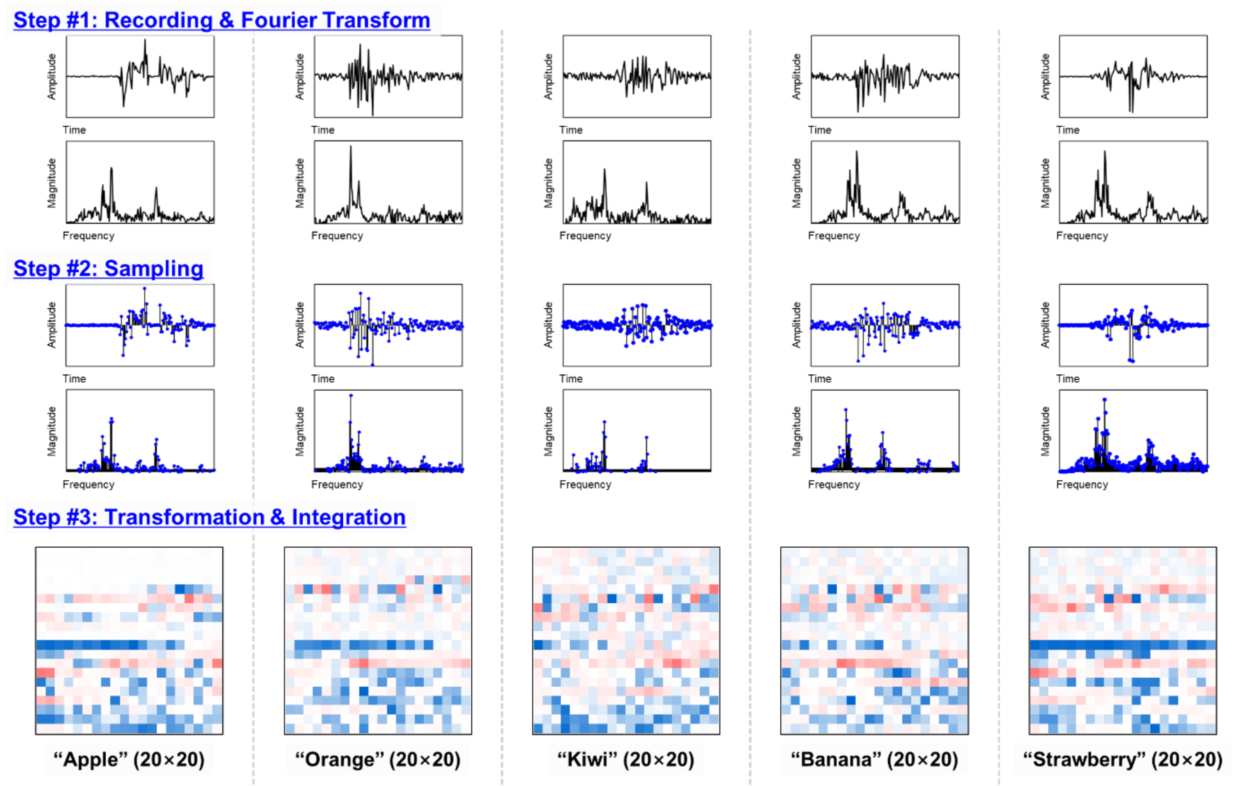

Supplementary Figure 23 | Design for the acoustic pattern and the designed acoustic patterns. See also Supplementary Note 15.

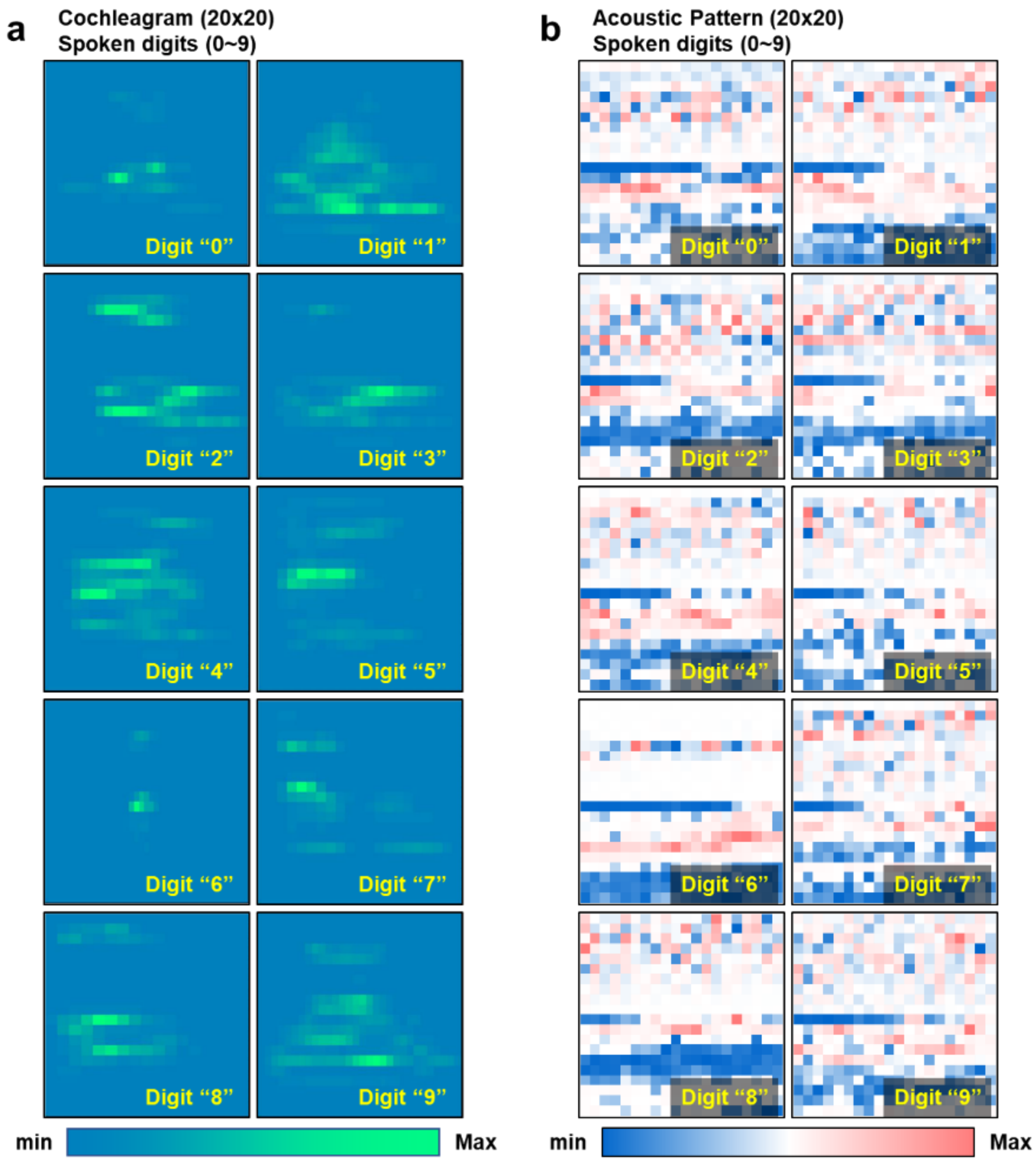

Supplementary Figure 24 | Datasets for the spoken digits. a,b, Datasets for the spoken digits (from “0” to “9”) as type of a cochleagram (20×20) (**a**) and type of an acoustic pattern (20×20) (**b**). See also Supplementary Note 16.

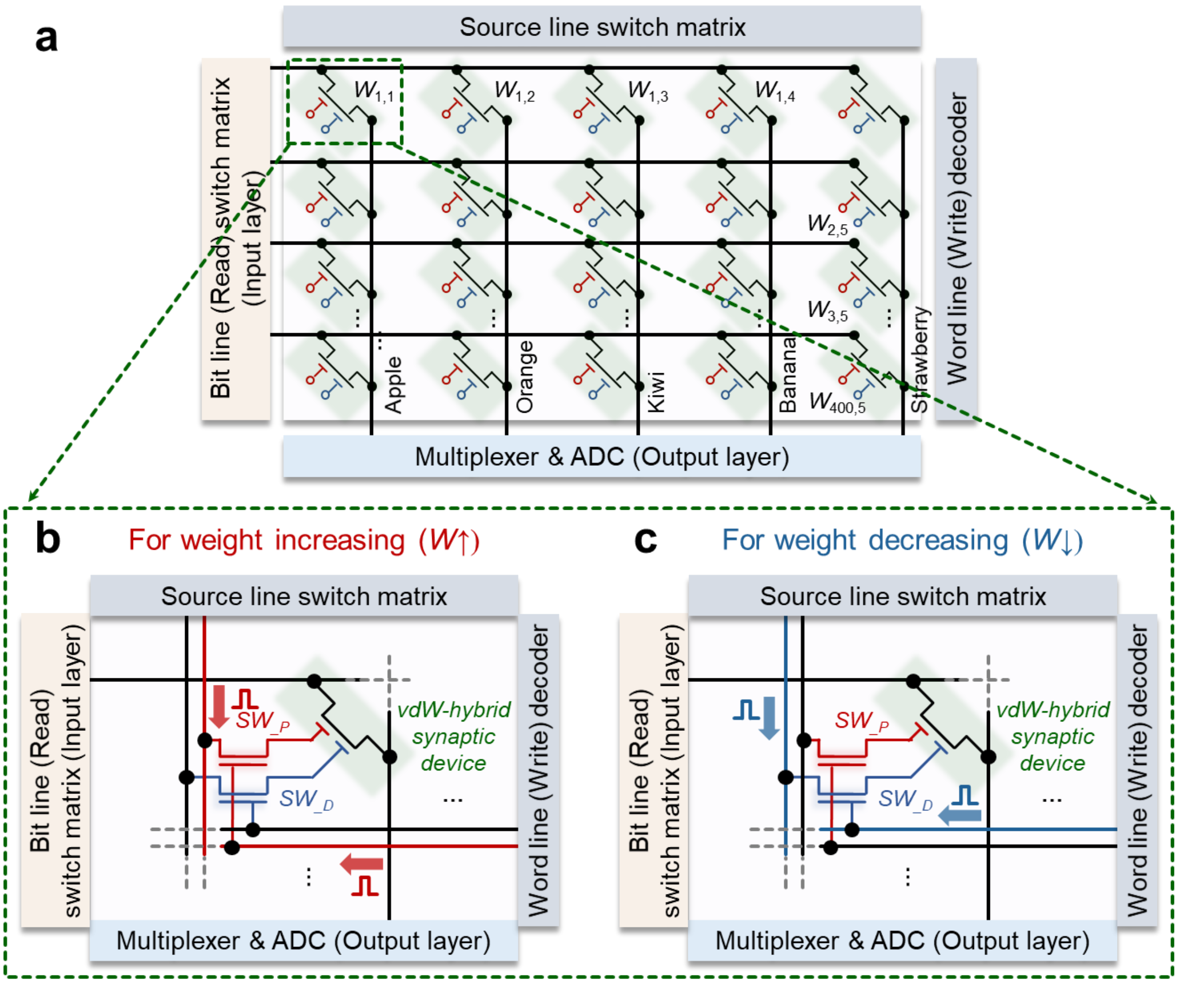

Supplementary Figure 25 | The hardware version structure to implement the conceptual neural network comprising the vdW-hybrid synaptic device. a, Schematic of the companion hardware version to the conceptual neural network comprising the vdW-hybrid synaptic device. **b,c,** Dynamics for weight increasing (**b**) and decreasing (**c**).

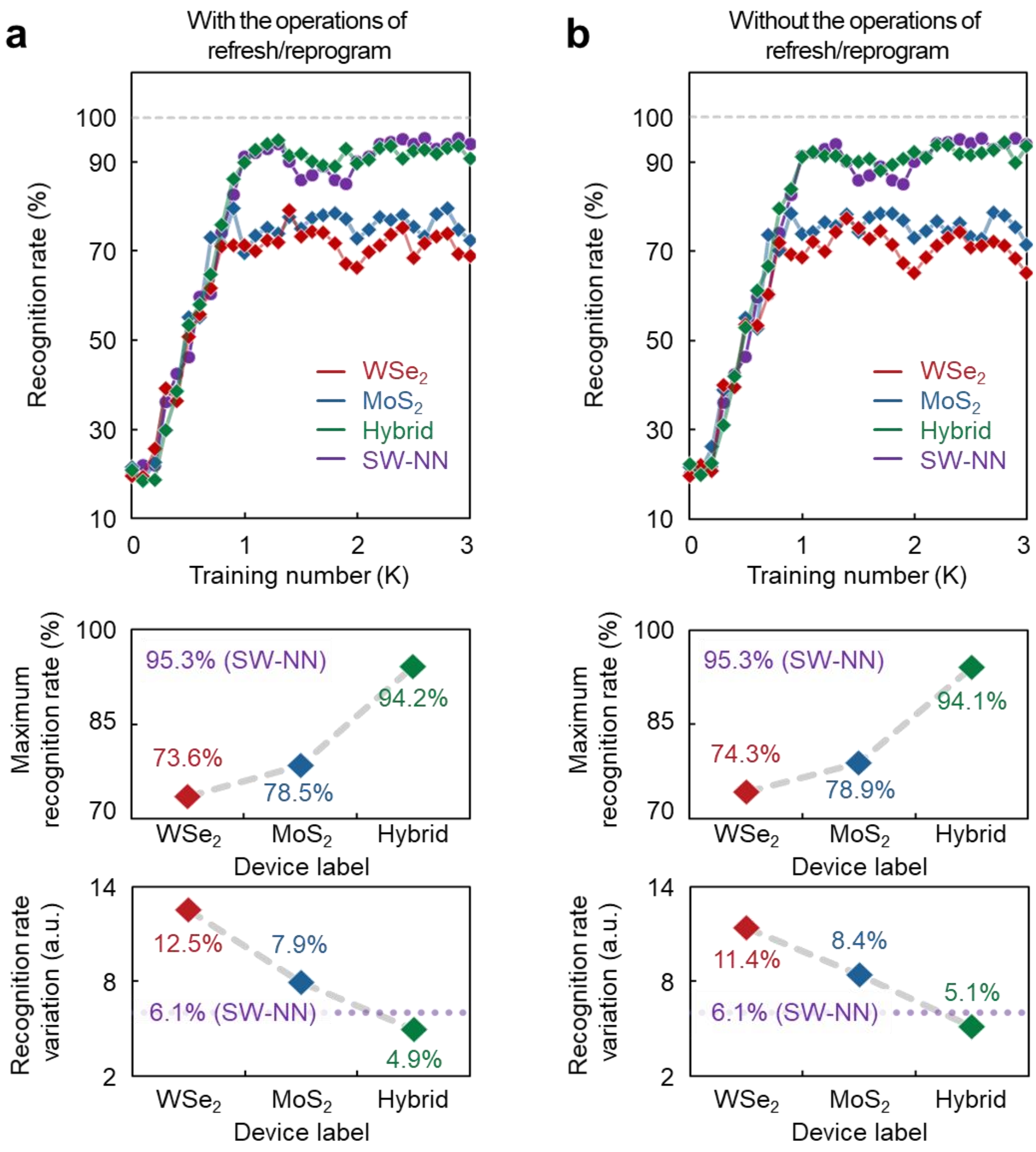

Supplementary Figure 26 | Acoustic pattern recognition rates and extracted maximum recognition rates and corresponding variations for the case with the operation of refresh/reprogram (a) and without the operation of refresh/reprogram (b).

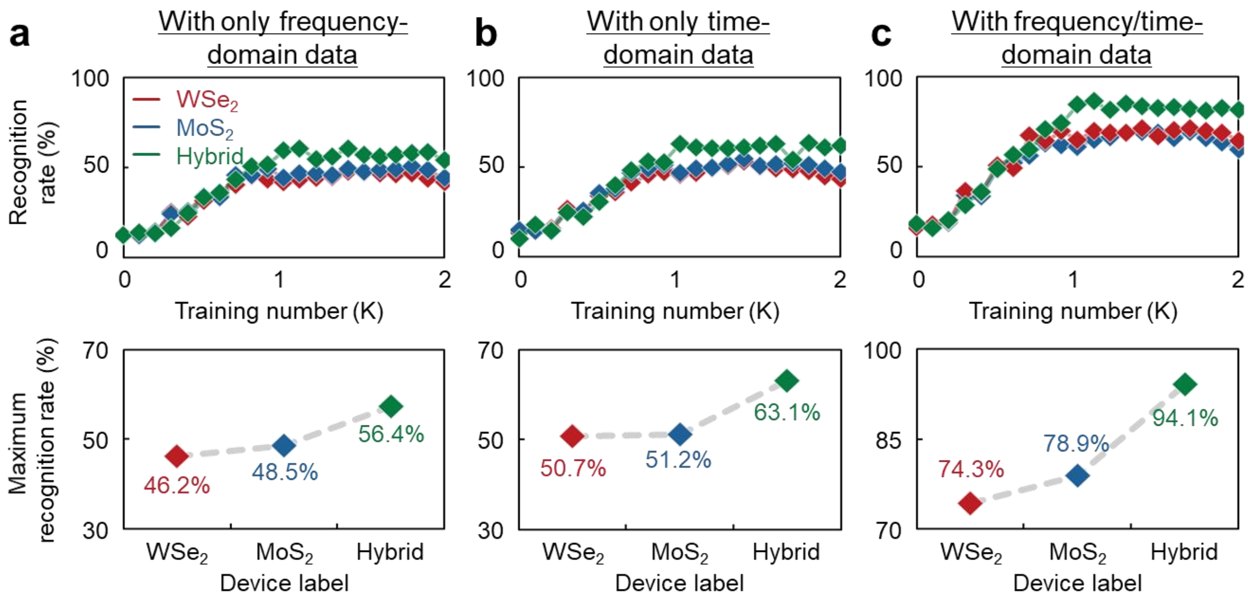

Supplementary Figure 27 | Recognition rates (top) and extracted maximum recognition rates (bottom) for the acoustic patterns (20×20) formed with time-domain data (a), with frequency-domain data (b), and with frequency/time-domain data (c).

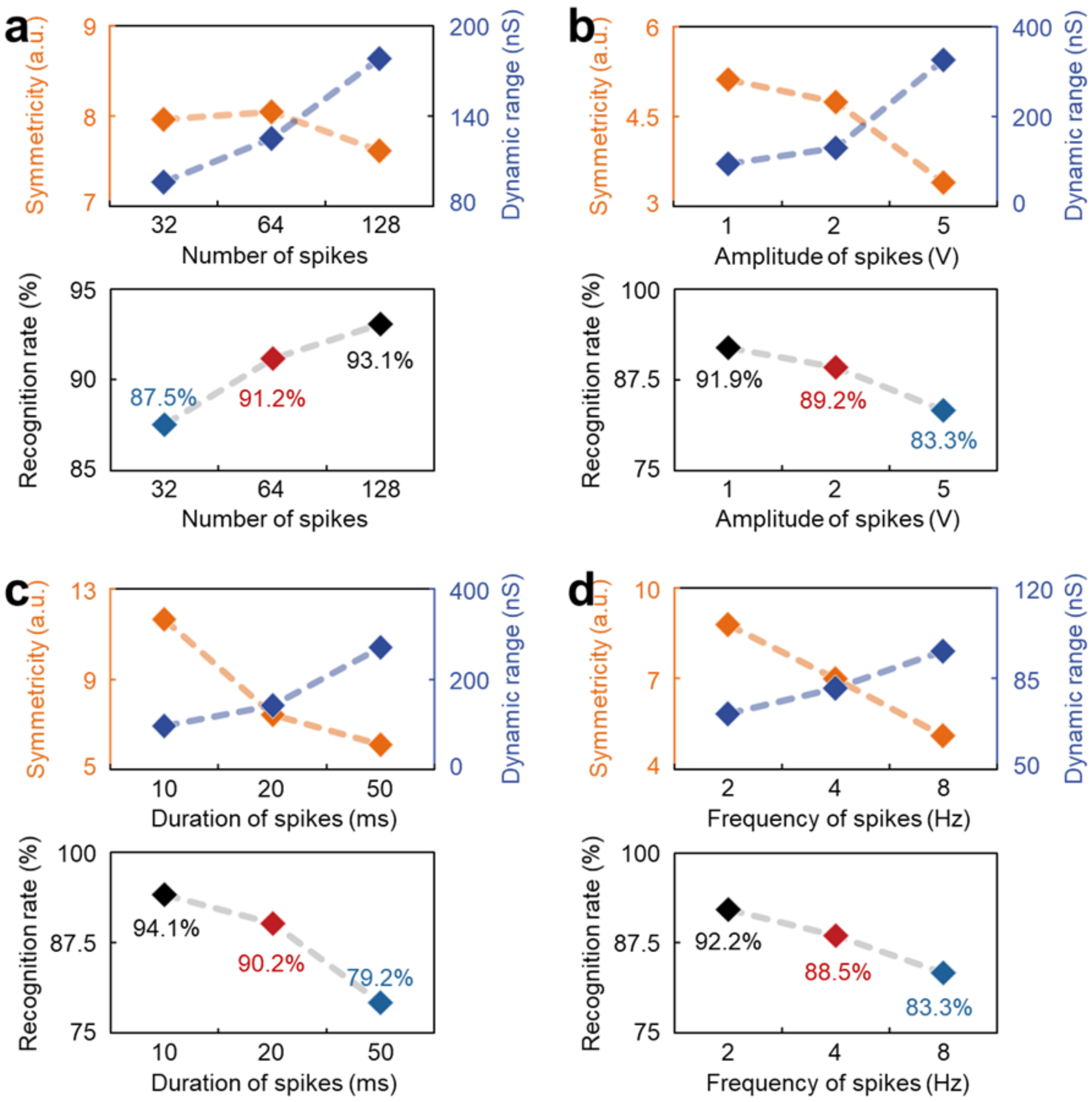

Supplementary Figure 28 | Recognition rate for acoustic patterns with respect to various spike conditions. a,b,c,d, Recognition rate with respect to number (**a**), amplitude (**b**), duration of spikes (**c**), and frequency of spikes (**d**). See also Supplementary Note 17.

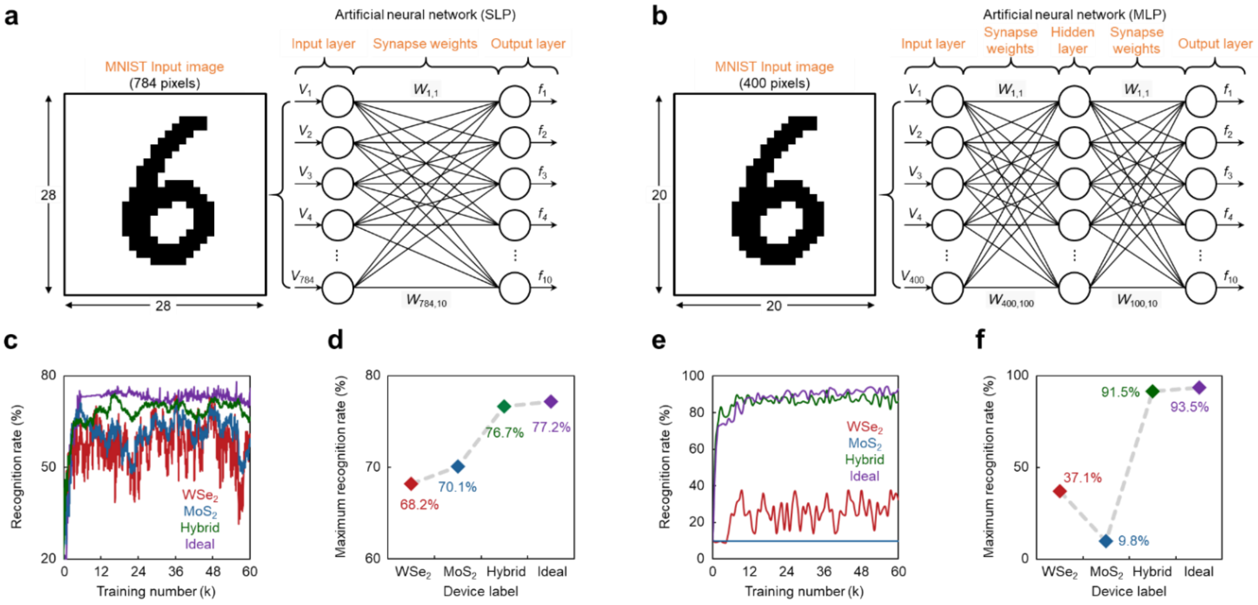

Supplementary Figure 29 | MNIST pattern recognition task. a, Single-layer neural network (NN) with 784 input neurons, 784 × 10 artificial synapses, and 10 output neurons for 28 × 28 MNIST pattern recognition. **b,** Multi-layer neural network with the layers of 400 input neurons, 400 × 100 and 100 × 10 artificial synapses, 100 hidden neurons, and 10 output neurons for 20 × 20 MNIST pattern recognition. **c,d,** Recognition rates based on single-layer NNs comprising WSe_2, MoS_2, hybrid, or ideal synaptic devices (see also Supplementary Table 3) (**c**), and corresponding maximum recognition rates (**d**). **e,f,** Recognition rates based on multi-layer NNs comprising WSe_2, MoS_2, hybrid, or ideal synaptic devices (**e**), and corresponding maximum recognition rates (**f**). See also Supplementary Note 18.

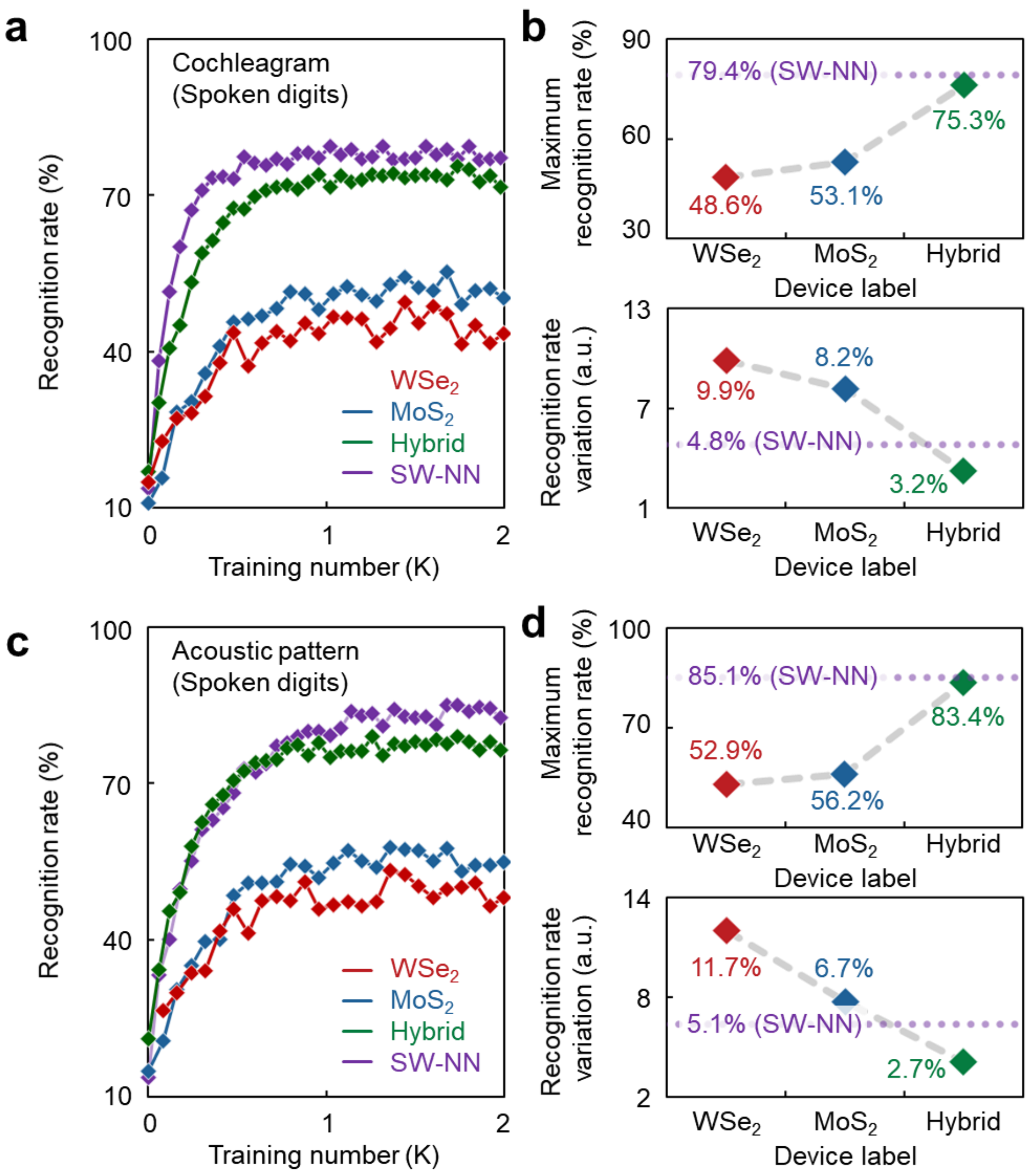

Supplementary Figure 30 | Training and inference simulation results for spoken digits datasets. a,b, Cochleagram pattern recognition rates (**a**), extracted maximum recognition rates, and corresponding variations (**b**). c,d, Acoustic pattern recognition rates (**c**), extracted maximum recognition rates, and corresponding variations (**d**).

	TaO_x/TiO_2	PCMO	Ag:α-Si	AlO_x/HfO_x	IFG-CBM	α-MoO_3	WSe_2	**This Work**
**Nonlinearity[10]**	0.4/0.63	3.68/6.76	2.4/4.88	1.96/0.61	~0.5/~0.5	-	2.3/2.4	**1.9 / 1.9**
**ON/OFF Ratio**	2	6.84	12.5	4.43	2	-	13.19	**14.03**
**Number of Conductance States**	102	50	97	40	50	-	100	**128**
**Cycle-to-Cycle Variation**	<1%	<1%	3.5%	5%	<1%	-	<1%	**<1%**
**R_{ON}**	5 MΩ	23 MΩ	26 MΩ	16.9 kΩ	-	1.4 MΩ	53.4 MΩ	**5 MΩ**
**Active area[a)]**	10^4 μm^2	150 × 150 nm^2	100 × 100 nm^2	400 × 400 nm^2	300 × 300 nm^2	5 × 5 μm^2	5 × 5 μm^2	**5 × 5 μm^2**
**Updating Energy[a)]**	-	-	-	-	-	0.16 pJ	0.53 pJ	**0.79 / 0.93 pJ**
**Retention**	-	>10^4 s	-	-	-	-	-	**>10^4 s**
**Endurance**	10^{12}	>10^9	>10^8	-	>10^8	-	-	**>10^5**
**Reference**	2	3	4	5	6	7	8	**-**

Supplementary Table 1 | Comparison of the vdW-hybrid synaptic device and other synaptic devices. The synaptic device in this work is compared with other synaptic devices reported heretofore, in terms of weight update linearity, on/off ratio, number of conductance states, cycle-to-cycle variation, R_{ON}, active area, updating energy, weight increase/decrease pulse, retention, and endurance. [a)]The energy will scale with active area. See also Supplementary Note 19.

	Ag:a-Si	TaO_x/HfO_x	PCMO	AlO_x/HfO_2	GST PCM	Epi-RAM	HZO FeFET	This work			Ideal
								**WSe_2**	**MoS_2**	**Hybrid**	
**Nonlinearity (P/D)**	2.4/−4.88	0.04/−0.63	3.68/−6.76	1.94/−0.61	0.105/2.4	0.5/−0.5	1.75/1.46	2.4/−8.5	5.5/−2.7	**1.95/−2.1**	0/0
**ON/OFF ratio**	12.5	10	6.84	4.43	19.8	50.2	45	12.32	3.13	**14.03**	>10
**# of conductance states**	97	128	50	40	100-120	64	32	128	128	**128**	>64
**Cycle-to-cycle variation**	3.5%	3.7%	<1%	5%	1.5%	2%	<0.5%	<1%	<1%	**<1%**	0%
**Online learning accuracy**	~72%	~80%	~33%	~20%	89%	92%	88%	37.1%	9.8%	**91.5%**	93.5%
**Reference**	4	2	3	5	9	11	12	**-**	-	-	-

Supplementary Table 2 | Comparison of vdW-hybrid synaptic device and other synaptic devices in terms of learning accuracy predicted by NeuroSim+ MNIST MLP simulator.[10]

	α_P	α_D	β_P	β_D	G_{max}	G_{min}
**Hybrid synaptic device**	2.75×10^{-9}	1.63×10^{-9}	1.9	1.9	122 nS	24 nS
**WSe_2 synaptic device**	2.01×10^{-9}	1.7×10^{-9}	2.3	21	95 nS	13 nS
**MoS_2 synaptic device**	2.43×10^{-9}	1.51×10^{-9}	8.6	2	109 nS	38 nS

Supplementary Table 3 | Extracted fitting parameters for pattern recognition tasks.

Supplementary Note 1. Nonlinearity analysis of the LTP/LTD curves

As shown in Supplementary Figure 3, the nonlinearity (β) was obtained by fitting the LTP/LTD curves with the following weight update formula:

$$G_{n+1} = G_n + \Delta G_P = G_n + \alpha_P e^{-\beta_P \frac{G_n - G_{min}}{G_{max} - G_{min}}},$$

$$G_{n+1} = G_n + \Delta G_D = G_n - \alpha_D e^{-\beta_D \frac{G_{max} - G_n}{G_{max} - G_{min}}}.$$

Here, G_{n+1} and G_n represent the conductance values of the synaptic device when the n+1th and nth spikes were applied, respectively. G_{max} and G_{min} represent the maximum and minimum conductance values. The parameters α and β indicate the step size of the conductance and the nonlinearity, respectively. As shown in Supplementary Figure 3a, a larger β corresponds to higher nonlinearity. The nonlinearity values obtained from the fitted LTP/LTD curves were 2.3/21 and 8.5/2, respectively (see Supplementary Fig. 3b). We also compared the nonlinearity of our hybrid device with the values of artificial synapses reported heretofore, as shown in Supplementary Figure 3c.[2-10]

Supplementary Note 2. Symmetricity analysis of the LTP/LTD curves

Supplementary Figure 4a shows the LTP/LTD curves of the vdW-hybrid synaptic device, as well as its kth (red dot) and (2n-k)th conductance states (blue dot). We defined the symmetricity as the reciprocal of the symmetric error (symmetricity = $\frac{1}{\text{symmetric error}}$). The symmetric error was defined as follows:

$$\text{Symmetric error} = \sum_{k=1}^{k=n} \frac{(G_N(k) - G_N(2n-k))^2}{n} = \sum_{k=1}^{k=n} \frac{((G(k) - G_{min}) - (G(2n-k) - G_{min}))^2}{n(G_{max} - G_{min})^2} = \sum_{k=1}^{k=n} \frac{(G(k) - G(2n-k))^2}{n(G_{max} - G_{min})^2}, \text{ where } G_N(k) = \frac{G(k) - G_{min}}{G_{max} - G_{min}}.$$

Here, G_N, G_{max}, and G_{min} represent the normalized value of the conductance, the maximum value of the conductance, and the minimum value of the conductance, respectively. Supplementary Figure 4b shows the asymmetric (symmetric error = ∞) and symmetric (symmetric error = 0) LTP/LTD curves.

Supplementary Note 3. Conductance variations for the three types of synaptic devices

As shown in Supplementary Figures 7a–c, we applied eight spikes in a row for potentiation and depression (two exhibitory, two inhibitory, two exhibitory, and two inhibitory) to the WCTs of the three types of devices. Consequently, the conductance of the WSe_2 and MoS_2 devices decreased (conductance variation σ = –19.65%) and increased (σ = +13.46%), respectively, compared with the initial values. For the hybrid synaptic device, a conductance similar to the initial value was observed under the same spike conditions (σ = –1.73%).

Supplementary Note 4. Energy distribution of trap states in WCL

The energy distribution of the trap states was investigated via micro photoluminescence (PL) and micro X-ray spectroscopy (XPS) measurements. As shown in Supplementary Figure 8, the PL spectra were measured on four different *h*-BN control samples without weight control layer (WCL), where temperature (T) and excitation wavelength (λ_{exc}) were 300 K and 325 nm, respectively.

We confirmed eight PL peaks which are expected to be caused by intrinsic defects such as a nitrogen

vacancy (V_N), a boron vacance (V_B), or an anti-site complex in which the nitrogen occupies the boron site (N_BV_N).[21] Then, as shown in Supplementary Figure 9, the PL spectra of the four h-BN samples with WCL (h-BN/WCL structure) were obtained under the same conditions (T = 300 K and λ_{ext} = 325 nm). Similarly to the control samples without WCL, in the four h-BN samples with WCL, we also confirmed the eight PL peaks. The intensity of the PL spectra increased overall, where the PL peaks 7&8 located at relatively large energy region increased significantly. Thus, it is expected that the energy levels for the trap states generated by the plasma treatment are mainly distributed in the higher energy region which is closer to the conduction band of h-BN.

As shown in Supplementary Figure 10, we then confirmed the increase in boron vacancy (V_B) and anti-site nitrogen vacancy (N_BV_N), where the atomic concentration of V_B and N_BV_N increased from 3.39% to 3.98%. Besides, new compounds with respect to carbon and fluorine were generated after the CF_4 plasma treatment. Thus, the energy levels of the newly generated traps seem to be related with the boron vacancies and/or compounds based on carbon and fluorine.

Supplementary Note 5. Electrical transfer curves of the potentiation and depression channels and their approximate uniform trap density in the WCL

Supplementary Figures 11a and d show the transfer curves of the potentiation (WSe_2) and depression (MoS_2) channels. We swept the control voltage from 15 to –15 V (for the potentiation channel) and from –15 to 15 V (for the depression channel) and then measured the postsynaptic current between the presynaptic and postsynaptic terminals. Without the WCL, hysteresis was not observed in either channel. This is mainly because a defect-free heterojunction was created between the h-BN and WSe_2/MoS_2 layers. In contrast, with the WCL, hysteresis was clearly observed. The WCLs were created via CF_4 plasma treatment with a 10-W reactive ion etcher power and 10-s process time.

In the LTP/LTD curves of the potentiation and depression channels, as shown in Supplementary Figures 11b and e, the control voltage shift (ΔV) between G_{min} and G_{max} was 1.6 (WSe_2) and 1.55 V (MoS_2), respectively. Assuming that the ΔV is determined only by ΔQ_i,

$$\Delta V = -\frac{\Delta Q_\mathrm{i}}{C_\mathrm{i}} = -\frac{1}{C_\mathrm{i}}\frac{1}{t_\mathrm{ox}}\int_0^{t_\mathrm{ox}} x\rho \, dx = -\frac{q n_\mathrm{avg} A}{2C_\mathrm{i} t_\mathrm{ox}}(t_\mathrm{ox}^2 - t_\mathrm{bn}^2),$$

where n_{avg} and A represents the approximate uniform trap density in the WCL layer and active area, respectively, and $t_\mathrm{ox} = t_\mathrm{bn} + t_\mathrm{wcl}$ (t_{bn} and t_{wcl} represent the thicknesses of the h-BN and WCL, respectively). C_i is defined as $C_\mathrm{bn} + C_\mathrm{wcl} = \frac{C_\mathrm{bn} C_\mathrm{wcl}}{(C_\mathrm{bn}+C_\mathrm{wcl})} = \frac{A\varepsilon_\mathrm{bn}\varepsilon_\mathrm{wcl}}{(\varepsilon_\mathrm{bn} t_\mathrm{wcl} + \varepsilon_\mathrm{wcl} t_\mathrm{bn})}$, where C_{bn} and C_{wcl} represent the capacitances of the h-BN and WCL, and ε_{bn} and ε_{wcl} represent the permittivity values for the h-BN and WCL.

Therefore, n_{avg} is obtained as

$$n_\mathrm{avg} = \frac{-2C_\mathrm{i} t_\mathrm{ox}\Delta V}{qA(t_\mathrm{ox}^2 - t_\mathrm{bn}^2)} \ [\mathrm{cm}^{-3}],$$

which is approximately 5.2×10^{17} and 5.8×10^{17} cm^{-3} for A = 25 μm^2 $\varepsilon_{bn} = 4.5\varepsilon_0$ and $\varepsilon_{wcl} = 4.8\varepsilon_0$, respectively (for the potentiation channel, t_{bn} = 60.1 and t_{wcl} = 11.1 nm; for the depression channel, t_{bn} = 64.1 and t_{wcl} = 10.5 nm).

Supplementary Note 6. Extraction of the injection barrier height in the potentiation and depression channel

As shown in Supplementary Figure 12, we found the barrier height in the potentiation and depression channel through temperature-dependent electrical measurements in the WSe_2 and MoS_2 transistor with Pt source/drain (for WSe_2) and Ti source/drain (for MoS_2) electrodes. Because the WSe_2 and MoS_2 transistor operates under thermionic emission and diffusion mechanisms, a conventional Richardson plot does not give a linear fit over the whole temperature range. Thus, we employed a modified Richardson plotting method based on a thermionic emission-diffusion model.[22-23] As shown in Supplementary Figure 12, the linearity in the $\ln(I_0/T^2) - q^2\sigma_0^2/2(k_BT)^2$ *versus* q/k_BT plot was obtained, where I_0 is the saturation current, T is the measurement temperature, q is the elementary charge, σ_0 is the standard deviation of the Gaussian distribution, and k_B is the Boltzmann constant. From the slope in the modified Richardson plot, the injection barrier height of potentiation and depression channel was estimated to be 0.31 and 0.19 eV, respectively.

Supplementary Note 7. PSC response to a negative voltage spike in the potentiation and depression channels

We investigated the negative voltage spike response in the current paths of the potentiation and depression channels in detail. When a negative voltage spike was applied to the WCT for each channel, the PSCs flowing through the potentiation and depression channels decreased from 68 to 46 nA and increased from 35 to 45 nA, respectively, as shown in Supplementary Figure 13a. This is because the de-trapping of the electrons from the WCL increased the number of electrons in the WSe_2 and MoS_2 channels, increasing and decreasing the width of the tunneling barrier (W_{TN}) from the presynaptic terminal (T_{pre}) metal to the TMDs (see Supplementary Fig. 13b).

Supplementary Note 8. Measurement setup for the KPFM analysis

The NX10 (Park Systems Corp.) AFM system was used to obtain the work function. Prior to the KPFM measurement, the KPFM tip (platinum/iridium (Pt/Ir)-coated Si tip) was calibrated on a highly oriented pyrolytic graphite (HOPG) surface. The HOPG surface is conventionally used to calibrate the work function of the KPFM tip owing to its clean surface and well defined work function (4.6 eV). The average ΔV_{CPD} values of the WSe_2 and MoS_2 flakes were obtained as –260 and –150 mV, respectively. As ΔV_{CPD} is the difference between the KPFM tip and sample work functions, the work function values of the WSe_2 and MoS_2 flakes can be calculated using the following equation: $\Phi_s = \Phi_{tip} - \Delta V_{CPD}$, where Φ_s and Φ_{tip} are the work functions of the samples (WSe_2 and MoS_2) and the KPFM tip, respectively. Here, Φ_{tip} was obtained from the sum of the HOPG work function (Φ_{HOPG}) and the ΔV_{CPD} between the KPFM tip and the HOPG surface ($\Phi_{tip} = \Phi_{HOPG} + \Delta V_{CPD\_HOPG}$). Therefore, the work function values of the WSe_2 and MoS_2 films can be estimated as approximately 4.86 and 4.75 eV, respectively (see Fig. 2d bottom).

Supplementary Note 9. The linearity of PSC determined by the weight updating origin and the polarity of channel

[Weight updating origin]

Electron trapping: When excitatory spikes are applied in a row to the WCT, the energy band of the semiconductor is instantly bent downward, inducing an electrical field that attracts electrons toward the WCL. Simultaneously, the probability of trap states being filled increases due to the upward movement of the semiconductor's Fermi level. Therefore, the trap sites at the WCL are partially filled with electrons for each excitatory spike, which gradually decreases the surface potential of the p- and n-type

semiconductors (# of holes in WSe_2 channel↑, # of electrons in MoS_2 channel↓).

Electron de-trapping: Meanwhile, when inhibitory spikes are applied in a row after the excitatory spikes, the energy band of the semiconductor is instantly bent upward, inducing an electrical field that de-traps electrons. Additionally, the many empty states in the semiconductor's conduction band allow the majority of trapped electrons to be released from the WCL during the initial few inhibitory spikes. Consequently, the surface potential of the semiconductors increases rapidly at first and then gradually becomes saturated (# of holes in WSe_2 channel↓, # of electrons in MoS_2 channel↑).

[Channel polarity]

WSe_2 (p-type) channel: After each excitatory spike is applied in a row to the WCT, the energy band of the WSe_2 semiconductor gradually moves down because of the trapped electrons, gradually increasing holes in the WSe_2 channel and the PSC. For the application of inhibitory spikes, the number of holes in the channel and the PSC decrease unevenly owing to the non-gradual de-trapping of electrons.
→ "Linear potentiation" and "Non-linear depression"

MoS_2 (n-type) channel: After each excitatory spike is applied in a row to the WCT, the energy band of the MoS_2 semiconductor gradually moves down like the above WSe_2 case because of the trapped electrons, gradually decreasing electrons in the MoS_2 channel and the PSC. For the application of inhibitory spikes, the number of electrons in the channel and the PSC increase unevenly owing to the non-gradual de-trapping of electrons.
→ "Linear depression" and "Non-linear potentiation"

Supplementary Note 10. The operating (reading and updating) energy of the vdW-hybrid synaptic device

As shown in Supplementary Figure 14, we investigated the reading/updating energy for the potentiation and depression channels, especially with respect to the amplitude and duration of spike. The reading/updating energy of vdW-hybrid synaptic device can be estimated using the following formula:

$$P_{\text{reading}} = V_{\text{spike}} \times T_{\text{duration}} \times I_{\text{PSC}}$$

$$P_{\text{updating}} = V_{\text{spike}} \times T_{\text{duration}} \times I_{\text{leakage}}$$

Here, V_{spike} and T_{duration} denote the amplitude and the duration of the spike, respectively. I_{PSC} and I_{leakage} denote the postsynaptic current across the hybrid channel and the gate leakage current flowing across the gate dielectric layer when a spike is applied, respectively.

Supplementary Note 11. PSC response with respect to the conditions of the spikes and CF_4 plasma treatment

We investigated the EPSC and IPSC responses with respect to the conditions of the spikes and CF_4 plasma treatment in the potentiation and depression channels in detail. When a positive voltage spike was applied to the WCTs for the potentiation and depression channels, the PSCs flowing through the potentiation and depression channels increased and decreased, respectively, as shown in Supplementary Figure 15a. As the amplitude and duration of the spikes increased, $\Delta G_{\text{Potentiation}}$ and $\Delta G_{\text{Depression}}$ in the potentiation and depression channels, respectively, increased (see Supplementary Fig. 15b and c). This is because the number of electrons trapped in the WCL increased with the amplitude and duration of the spikes, inducing a larger width change in the tunneling barrier from the presynaptic terminal metal to the TMDs. As illustrated in Supplementary Figure 15d, we also varied the conditions of the CF_4 plasma treatment, i.e., the process time and the reactive ion etcher power. As the plasma process time and the reactive ion etcher power increased, $\Delta G_{\text{Potentiation}}$ and $\Delta G_{\text{Depression}}$ in the potentiation and depression

channels, respectively, increased, as shown in Supplementary Fig. 15e and f. This is presumably because the number of trap states in the WCL increased with the process time and power.

Supplementary Note 12. Nonlinearity with respect to the conditions of spikes and CF_4 plasma treatment

As shown in Supplementary Figure 18, we investigated the nonlinearity in the potentiation channel and depression channel with respect to the conditions of spikes and CF_4 plasma treatment. When a larger or longer spike was applied to the WCT, the nonlinearity increased, as shown in Supplementary Figures 18a-b. When spikes were applied, the corresponding current was abruptly increased and then slowly decreased. This is because the electrons trapped in shallow/fast traps were recovered transiently. On the other hand, as shown in Figures 18c-d, it was confirmed that the nonlinearity was maintained regardless of the plasma treatment conditions.

Supplementary Note 13. Cycle-to-cycle variation (CCV) of the synaptic devices

The CCV represents the degree of variation during the conductance potentiation or depression process. To estimate the CCV, we employed the following weight update formula:

$$G_{n+1} = G_n + \Delta G_P = G_n + \sigma_P \alpha_P e^{-\beta_P \frac{G_n - G_{min}}{G_{max} - G_{min}}},$$

$$G_{n+1} = G_n + \Delta G_D = G_n - \sigma_D \alpha_D e^{-\beta_D \frac{G_{max} - G_n}{G_{max} - G_{min}}},$$

where $\sigma_P = \mathrm{CCV_P} \times \mathrm{Randn}$ and $\sigma_D = \mathrm{CCV_D} \times \mathrm{Randn}$.

Here, σ_P and σ_D are parameters related to the CCVs in the potentiation and depression channels, respectively. σ is defined as the product of the CCV and Randn, and Randn is a normalized random number ranging from -1 to +1. Supplementary Figures 20a and b show the fitted potentiation and depression curves, respectively, for different CCVs (0.1%, 3%, and 5%), which were obtained using the above formula. As a result of fitting the LTP and LTD curves of the hybrid synaptic device, a CCV of 0.1% was obtained for the potentiation and depression channels, respectively, as shown in Supplementary Figure 20c.

Supplementary Note 14. Dependence of dynamic range and symmetricity on spike conditions

For the dynamic range of LTP/LTD curves, when a larger, longer, or faster spike was applied to the WCT, this value increased, as shown in Figures 3d-f. It is predicted that the greater number of electrons trapped by spikes with larger amplitude, duration, and frequency induced higher concentration of holes in the WSe_2 and MoS_2 channels, as shown in Supplementary Figures 22a and b.

For the symmetricity of LTP/LTD curves, when a larger, longer, or faster spikes was applied to the WCT, this value decreased, as shown in Figures 3d-f. As illustrated in Figures 2e and f and Supplementary Figure 12a, when an exhibitory spike was applied, the corresponding current was abruptly increased and then slowly decreased. This is because the electrons trapped in shallow/fast traps were recovered transiently. When high-frequency spikes are applied in a row, electrons are trapped consecutively before the electrons are sufficiently released from the WCL, as shown in Supplementary Figure 22c. Therefore, at the end of LTP or LTD process, the number of unoccupied traps at the WCL is reduced. This

consequently increases nonlinearity for LTP/LTD curves and reduces symmetricity. In addition, similar dependence of nonlinearity and symmetricity is expected when spikes with high-amplitude and duration are applied in a row.

Supplementary Note 15. Design for the acoustic pattern and the designed acoustic patterns

For the simulation task shown in Supplementary Figure 23, we prepared training and inference datasets with 3000 training and 400 inference acoustic pattern images for five distinct words: “apple,” “orange,” “kiwi,” “banana,” and “strawberry.” The dataset for each word consisted of 600 training and 80 inference acoustic pattern images.

Supplementary Note 16. Datasets for spoken digits

As shown in Supplementary Figure 24, we prepared two types of spoken digit datasets consisting of cochleagram patterns or our acoustic patterns, where the Lyon’s auditory model was applied to create the cochleagram patterns.[24] Datasets consisted of 2000 training and 2000 inference pattern images for ten distinct digits (from “0” to “9”), and each pixel in the pattern images had a grayscale value in range of 0-255.

Supplementary Note 17. Recognition rate for acoustic patterns with respect to various spike conditions

As shown in Supplementary Figure 28, we investigated the recognition rate for acoustic patterns with respect to various spike conditions (number, amplitude, duration, and frequency of spikes). As the number of spikes increases (from 32 to 128), the recognition rate increased (from 87.5 to 93.1%). This is mainly because the dynamic range increases (from 96.12 to 178 nS) while the symmetricity is maintained (between 7.95 and 7.61) as the number of spikes increases. On the other hand, as the amplitude, duration, and frequency of spikes increase, the recognition rate decreases (amplitude: from 93.1 to 78.5%, duration: from 93.6 to 82.5%, frequency: from 90.7 to 81.6%). Here, the dynamic range increases as the amplitude (from 95 to 326 nS), duration (from 91 to 270 nS), and frequency (from 71 to 95 nS) of spikes increase, and the symmetricity decreases (amplitude: from 91.9 to 83.3%, duration: from 94.1 to 79.2%, frequency: from 92.2 to 83.3%). In summary, (*i*) the increase in the number of spikes and the decrease in the amplitude, duration, and frequency of spikes induce an increase in the recognition of HW-NN, and (*ii*) the sensitivity of the recognition rate of HW-NN on symmetricity is larger than that on dynamic range.

Supplementary Note 17. MNIST pattern recognition task

Supplementary Figure 29 shows the MNIST pattern recognition tasks via single-layer NNs and multi-layer NNs, where they comprised artificial synapses such as WSe_2, MoS_2, hybrid, or ideal synaptic devices. For the single-layer NNs with 784 input neurons, 784 × 10 artificial synapses, and 10 output neurons, the voltage signals corresponding to each pixel of MNIST pattern were assumed to be applied to the input neuron layer. Those were multiplied by the synapses weight ($W_{n,m}$) and then summed at the output neurons, resulting in output currents. The output value obtained via the sigmoid activiation function was then compared with each label value. Finally, synapse weights were updated on basis of

backpropagation algorithm (described in Experimental Section). The maximum recognition rates were 68.2%, 70.1%, 76.7%, and 77.2% for the WSe_2, MoS_2, vdW-hybrid, and ideal synaptic devices (see also Supplementary Table 3).

For the multi-layer NNs with 400 input neurons, 400×100 and 100×10 artificial synapses, and 10 output neurons, we employed NeuroSim+, which is an integrated simulation framework for benchmarking synaptic devices in terms of the system-level learning accuracy.[11] The maximum recognition rates were 37.1%, 9.8%, 91.5%, and 93.5% for the WSe_2, MoS_2, vdW-hybrid, and ideal synaptic devices. Here, ideal synaptic devices feature linear and symmetric conductance change, >10 dynamic range, >128 number of conductance states, and <1% cycle-to-cycle variation, as shown in Supplementary Table 2.

Supplementary Note 19. Evaluation of nonlinearity of the artificial synapses reported to date

As shown in Supplementary Table 1, the nonlinearity of the artificial synapses reported to date was evaluated. Here, the evaluation of the nonlinearity was performed by fitting the LTP/LTD characteristic curves with the following weight-update formulas:

$$G_{\mathrm{n+1}} = G_{\mathrm{n}} + \Delta G_{\mathrm{P}} = G_{\mathrm{n}} + \alpha_{\mathrm{P}} e^{-\beta_{\mathrm{P}}\frac{G_{\mathrm{n}}-G_{\mathrm{min}}}{G_{\mathrm{max}}-G_{\mathrm{min}}}},$$

$$G_{\mathrm{n+1}} = G_{\mathrm{n}} + \Delta G_{\mathrm{D}} = G_{\mathrm{n}} - \alpha_{\mathrm{D}} e^{-\beta_{\mathrm{D}}\frac{G_{\mathrm{max}}-G_{\mathrm{n}}}{G_{\mathrm{max}}-G_{\mathrm{min}}}}.$$

In these equations, $G_{\mathrm{n+1}}$ and G_{n} denote the synaptic conductance when the n+1th and nth pulses are applied, respectively. G_{max} and G_{min} indicate the maximum and minimum conductance values, respectively. Parameters α and β are the conductance change amount and the nonlinearity, respectively.

Supplementary References

1. Saito, R. Raman spectroscopy of transition metal dichalcogenides. *J. Phys.: Condens. Matrer*. **28**, 353002 (2016).

2. Wang, Y. -F. et al. Characterization and Modeling of Nonfilamentary Ta/TaO_x/TiO_2/Ti Analog Synaptic Device. *Sci. Rep.* **5**, 10150 (2015).

3. Park, S. et al. Neuromorphic speech systems using advanced ReRAM-based synapse. *2013 IEDM*. (2013).

4. Jo, S. H. et al. Nanoscale Memristor Device as Synapse in Neuromorphic Systems. *Nano Letters*. **10**, 1297-1301 (2010).

5. Woo, J. et al. Improved Synaptic Behavior Under Identical Pulses Using AlO_x/HfO_2 Bilayer RRAM Array for Neuromorphic Systems. *IEEE Electron Device Letters*. **37**, 994-997 (2016).

6. Fuller, E. J. et al. Parallel programming of an ionic floating-gate memory array for scalable neuromorphic computing. *Science*. **364**, 570-574 (2019).

7. Yang, C. S. et al. A Synaptic Transistor based on Quasi-2D Molybdenum Oxide. *Adv. Mater.* **29**, 1700906 (2017).

8. Seo, S. et al. Artificial optic-neural synapse for colored and color-mixed pattern recognition. *Nat. Commu.* **9**, 5106 (2018).

9. Kuzum, D. et al. Nanoelectronic programmable synapses based on phase change materials for brain-inspired computing. *Nano Letters.* **12**, 2179-2186 (2011).

10. Chen, P.-Y. et al. NeuroSim: A circuit-level macro model for benchmarking neuro-inspired architectures in online learning. *IEEE T. Compt. Aid. D.* **37**, 3067-3080 (2018).

11. Choi, S. et al. SiGe epitaxial memory for neuromorphic computing with reproducible high performance based on engineered dislocations. *Nat. Mat.* **17**, 335-340 (2018).

12. Jerry, M. et al. Ferroelectric FET analog synapse for acceleration of deep neural network training. *2017 IEDM.* (2017).

13. Xu, R. et al. Vertical MoS_2 double-layer memristor with electrochemical metallization as an atomic-scale synapse with switching thresholds approaching 100 mV. *Nano Letter*. **19**, 2411-2417 (2019).

14. Li, D. et al. MoS_2 memristors exhibiting variable switching characteristics toward biorealistic synaptic emulation. *ACS Nano*. **12**, 9240-9252 (2018).

15. Yan, X. et al. A new memristor with 2D $Ti_3C_2T_X$ MXene flakes as an artificial bio-synapse. *Small*. **15**, 1900107 (2019).

16. Tian, H. et al. Anistropic black phosphorus synaptic device for neuromorphic application. *Adv. Mat.* 28, 4991-4997 (2016).

17. Chen, J. et al. Time-tailoring van der Waals heterostructures for human memory system programming. *Adv. Sci.* **6**, 1901072 (2019).

18. Sun, J. et al. Optoelectronic synapse based on IGZO-Alkylated graphene oxide hybrid structure. *Adv. Funct. Mat.* **28**, 1804397 (2018).

19. Mao, J.-Y. et al. Artificial synapses emulated through a light mediated organic-inorganic hybrid transistor. *J. Mat. Chem. C.* **7**, 48-59 (2019).

20. Jang, E.-K., Park, Y. & Lee, J.-S. Reversible uptake and release of sodium ions in layered SnS2-reduced graphene oxide composites for neuromorphic devices. *Nanoscale*. **11**, 15382-15388 (2019).

21. Tran, T. T. et al. Quantum emission from hexagonal boron nitride monolayers. *Nat. Nanotech.* **11**, 37-41 (2016).

22. Hastas, N. A. et al. Electrical transport and low frequency noise characteristics of Au/n-GaAs Schottky diodes containing InAs quantum dots. *Semicon. Sci. and Tech.* **19**, 461-467 (2004).

23. Lin, Y.-F. et al. Barrier inhomogeneities at vertically stacked graphene-based heterostructures. *Nanoscale.* **6**, 795-799 (2014).

24. Lyon, R. F. et al. A Computational Model of Filtering, Detection, and Compression in the Cochlea. *Proceed. of IEEE-ICASSP-82.* 1282-1285 (1982).
